# Cancer Patient Experience of Uncertainty While Waiting for Genome Sequencing Results

**DOI:** 10.3389/fpsyg.2021.647502

**Published:** 2021-04-22

**Authors:** Nicci Bartley, Christine E. Napier, Zoe Butt, Timothy E. Schlub, Megan C. Best, Barbara B. Biesecker, Mandy L. Ballinger, Phyllis Butow

**Affiliations:** ^1^Psycho-Oncology Co-operative Research Group, Faculty of Science, School of Psychology, The University of Sydney, Sydney, NSW, Australia; ^2^Cancer Theme, Garvan Institute of Medical Research, Sydney, NSW, Australia; ^3^Faculty of Medicine and Health, Sydney School of Public Health, The University of Sydney, Sydney, NSW, Australia; ^4^Institute for Ethics & Society, The University of Notre Dame Australia, Sydney, NSW, Australia; ^5^Research Triangle Institute International, Bethesda, MD, United States; ^6^St Vincent's Clinical School, University of NSW, Sydney, NSW, Australia

**Keywords:** uncertainty, genome sequecing, cancer, psychosocial, genomic, genetic testing, anxiety

## Abstract

There is limited knowledge about cancer patients' experiences of uncertainty while waiting for genome sequencing results, and whether prolonged uncertainty contributes to psychological factors in this context. To investigate uncertainty in patients with a cancer of likely hereditary origin while waiting for genome sequencing results, we collected questionnaire and interview data at baseline, and at three and 12 months follow up (prior to receiving results). Participants (*N* = 353) had negative attitudes towards uncertainty (*M* = 4.03, SD 0.68) at baseline, and low levels of uncertainty at three (*M* = 8.23, SD 7.37) and 12 months (*M* = 7.95, SD 7.64). Uncertainty about genome sequencing did not change significantly over time [*t*(210) = 0.660, *p* = 0.510]. Greater perceived susceptibility for cancer [*r*(348) = 0.14, *p* < *0*.01], fear of cancer recurrence [*r*(348) = 0.19, *p* < *0*.01], perceived importance of genome sequencing [*r*(350) = 0.24, *p* < *0*.01], intention to change behavior if a gene variant indicating risk is found [*r*(349) = 0.29, *p* < *0*.01], perceived ability to cope with results [*r*(349) = 0.36, *p* < *0*.01], and satisfaction with decision to have genome sequencing [*r*(350) = 0.52, *p* < *0*.01] were significantly correlated with negative attitudes towards uncertainty at baseline. Multiple primary cancer diagnoses [B = −2.364 [−4.238, −0.491], *p* = 0.014], lower perceived ability to cope with results [B = −0.1.881 [−3.403, −0.359], *p* = 0.016] at baseline, greater anxiety about genome sequencing (avoidance) [B = 0.347 [0.148, 0.546], *p* = 0.0012] at 3 months, and greater perceived uncertainty about genome sequencing [B = 0.494 [0.267, 0.721] *p* = 0.000] at 3 months significantly predicted greater perceived uncertainty about genome sequencing at 12 months. Greater perceived uncertainty about genome sequencing at 3 months significantly predicted greater anxiety (avoidance) about genome sequencing at 12 months [B = 0.291 [0.072, 0.509], *p* = 0.009]. Semi-structured interviews revealed that while participants were motivated to pursue genome sequencing as a strategy to reduce their illness and risk uncertainty, genome sequencing generated additional practical, scientific and personal uncertainties. Some uncertainties were consistently discussed over the 12 months, while others emerged over time. Similarly, some uncertainty coping strategies were consistent over time, while others emerged while patients waited for their genome sequencing results. This study demonstrates the complexity of uncertainty generated by genome sequencing for cancer patients and provides further support for the inter-relationship between uncertainty and anxiety. Helping patients manage their uncertainty may ameliorate psychological morbidity.

## Introduction

Cancer is a distressing experience (Foster et al., 2009; Montgomery and McCrone, 2010). Uncertainties about diagnosis and prognosis are major factors that influence patients' cancer experience (Mishel et al., 1984). Uncertainty can change over time but is typically highest at diagnosis when the patient lacks an understanding of their illness. Yet it can also increase when patients undergo scans or tests related to their cancer. Receiving a cancer diagnosis at a younger age (Kornblith et al., 2007) or receiving multiple cancer diagnoses (Thong et al., 2013), can intensify uncertainty. This cascade of uncertainty may include additional uncertainties about the origins of the cancer(s), including whether the cancer risk may have been inherited, and have implications for relatives (Bartley et al., 2021). Intolerance of uncertainty has been associated with poorer psychological outcomes in cancer patients (Kurita et al., 2013).

Patients who are experiencing illness-related uncertainty may seek out information to reduce their uncertainty (Mishel, 1988). For patients with a cancer that is likely due to inherited risk, information seeking may include undergoing genomic testing, including, genome sequencing to determine if they have a gene variant that would indicate a heritable origin for their cancer and/or provide information about their future risk of cancer or another illness.

The complexity of genomics is likely to introduce multiple uncertainties (Han et al., 2017) for patients, such as the scientific uncertainty (i.e., whether genome sequencing will provide disease information), practical uncertainty (i.e., lack of knowledge about processes), and personal uncertainty (i.e., psychosocial effects and implications of results for patients and relatives). A systematic review of studies investigating the patient experience of uncertainty in cancer genomics (Bartley et al., 2020b) concluded that while patients approach genomic testing as a strategy to reduce illness-related uncertainties, genomics may not reduce them. Previous research found that penetrance of a pathogenic variant (moderate vs. high) rather than type of genomic result (positive or negative) influenced patient perception of uncertainty in the cancer hereditary risk context (Lumish et al., 2017; Esteban et al., 2018). However, aambiguity surrounding the meaning of a variant of uncertain significance (VUS) result can create uncertainties about future risk for participants (Solomon et al., 2017). In the treatment context, regardless of the genomic result (positive or negative), participant treatment decision uncertainty was reduced (Holt et al., 2013; Levine et al., 2016). How patients appraise uncertainties, both negatively and positively, is important in how the patient experiences uncertainty, as well as specifying the coping strategies they utilize to manage the uncertainties (Biesecker et al., 2014; Bartley et al., 2020b).

The published literature on uncertainty in cancer genomics has been largely cross-sectional (Braithwaite et al., 2002; Pellegrini et al., 2012; Leventhal et al., 2013; Hitch et al., 2014; Lumish et al., 2017; Solomon et al., 2017), or when longitudinal (Holt et al., 2013; Bradbury et al., 2015; Levine et al., 2016; Esteban et al., 2018) focused on uncertainties at the time of choosing to undergo genomic testing and immediately following receipt of results. Where patients can afford genome sequencing in a clinical context, they will wait ~16 weeks for their results (Victorian Clinical Genetic Services, 2019). Patients who meet eligibility criteria can access testing within research studies without meeting the specific requirements needed in an Australian clinical context. In this context, genome sequencing is conducted at reduced or no cost (Bartley et al., 2020a). However, the wait of genome sequencing results can be much longer for patients due to the research nature of the study which does not have the same timeline imperatives as a clinical service. There is little known about the experience of managing uncertainty while awaiting results, and the potential psychological impact of prolonged uncertainty about testing for cancer patients. Exploring the uncertainties that patients experience while waiting for genome sequencing results will provide clinicians with a greater understanding of the sources and types of uncertainties that their patients face. Previous research (Bartley et al., 2021) has provided insights into the cancer patients' experience of uncertainty when agreeing to undertake genome sequencing. The aim of this study was to understand the cancer patients' experience of uncertainties while waiting for genome sequencing results, including the influence of attitudes toward uncertainty on the degree of perceived uncertainty about genome sequencing, changes in uncertainty over time, and the psychological outcomes of the uncertainty.

These patients were undertaking genome sequencing to contribute to research and potentially obtain more information about their cancer risk, a circumstance wrought with uncertainty. Further, engaging in genomics research where results are returned has the potential to generate additional practical, personal and scientific uncertainties (Han et al., 2017). As such, we hypothesized that perceptions of uncertainty about genome sequencing would increase with time while participants awaited novel genomic information which they hoped would reduce their illness uncertainty.

Patient appraisal of uncertainty, as well as the coping strategies utilized to manage uncertainties are important in how patients experience uncertainties (Biesecker et al., 2014; Bartley et al., 2020b). Previous qualitative research with this cohort (Bartley et al., 2021) found that participants who had negative attitudes towards uncertainty experienced ongoing uncertainties throughout the 2–4 weeks following agreeing to pursue genome sequencing. The decision to undertake genome sequencing did not reduce their illness or genome sequencing related uncertainty, as they were still waiting for genome sequencing results. We therefore anticipated that negative attitudes towards uncertainty at baseline would predict greater perceived uncertainty about genome sequencing at 12-month follow-up. As intolerance of uncertainty has also been linked to poorer psychological outcomes in cancer patients, we anticipated that negative attitudes towards uncertainty at baseline would also predict worse psychological outcomes (greater fear of cancer recurrence, higher anxiety, more depressive symptoms, and greater distress) at 12 months.

## Materials and Methods

The research presented is part of a longitudinal mixed methods psychosocial study: Psychosocial issues in Genomic Oncology (PiGeOn) study (Best et al., 2018). The PiGeOn study is a sub-study of an Australia-wide genomic study being conducted at the Garvan Institute of Medical Research, the Genetic Cancer Risk in the Young (RisC) study. The RisC study aims to identify clinically actionable, pathogenic gene variants that likely contribute to the development of cancer at an early age, and/or to multiple cancers at any age. Adults who have had a cancer diagnosis (other than non-melanoma skin cancer) under the age of 40, or two primary cancer diagnoses at an age younger than 50 years, or three primary cancer diagnoses at any age, are eligible to participate in the RisC study. RisC participants provide a blood sample on which genome sequencing is performed. Participants did not undergo pre-test genetic counseling prior to providing a blood sample for genome sequencing. A researcher provided participants with written information about genome sequencing, and potential results (Supplementary Material—PICF), and participants were given the opportunity to ask questions as part of the consent process. Participants choose whether they would like to be informed if they have a pathogenic variant that increases the likelihood of cancer and/or are found to have a secondary finding that may be important to their health, in accordance with the recommendations of the American College of Medical Genetics and Genomics and the Association for Molecular Pathology for reporting secondary findings in clinical exome and genome sequencing (Kalia et al., 2017). Variants of uncertain significance are not returned to participants in this study. If participants are found to carry a pathogenic gene variant, they are referred to a genetic counsellor. If the variant is cancer-related, participants are offered a tailored risk management plan through the Surveillance in Multi-Organ Cancer-prone syndromes study.

Participants consent to the PiGeOn study at the same time they consent to the RisC study. The PiGeOn study is investigating the psychosocial implications of genome sequencing by administering questionnaires and semi-structured interviews (with a subset of participants) completed at baseline (T0, within 1 month of consenting to genome sequencing) and 3 (T1) and 12-month (T2) follow-up. All PiGeOn study data is collected prior to patients receiving results. This manuscript reports on cancer patients' experiences of uncertainties over a 1-year period, while awaiting genome sequencing results.

### Questionnaire

Psychometrically validated scales were used where available, supplemented by adapted scales or study-developed items when validated scales were unavailable (Best et al., 2018). In addition to demographic and patient disease characteristics, the study questionnaire included measures of knowledge of and attitudes towards and levels of genome sequencing, and psychosocial outcomes (Table 1, Supplementary Material—PiGeOn RisC T0 questionnaire; Supplementary Material—PiGeOn RisC T1 questionnaire; Supplementary Material—PiGeOn RisC T2 questionnaire). The Attitude towards Uncertainty Scale (Braithwaite et al., 2002) was administered at baseline (T0). Level of uncertainty about genome sequencing was measured at 3 months (T1) and 1 year (T2) follow up with the adapted Multidimensional Impact of Cancer Risk Assessment (MICRA) Uncertainty sub-scale (Cella et al., 2002). The MICRA was developed to measure the specific impact of results disclosure after genetic testing. As PiGeOn participants were required to wait ~12 months for their genome sequencing results, our focus was on assessing the impact of waiting for results in this cohort. At the time of designing this study, there were no previously developed scales which specifically measured distress in this context, we adapted the MICRA for this purpose. For example, were the original MICRA item was “Having problems enjoying my life because of my test result” this was adapted to “Having problems enjoying my life while waiting for my test result.”

**Table 1:** Description of quantitative study measures and assessment time points.

**Measure**	**Baseline (T0)**	**3-month follow-up (T1)**	**12-month follow-up (T2)**
**Demographic characteristics** included age, sex, marital status, socio-economic status (SES) and accessibility/remoteness (ARIA) determined by postcode, educational level, country of birth, language spoken at home, number of biological children, smoking status, alcohol consumption, occupation.	X		
**Clinical characteristics** included personal cancer history (diagnosis and date of diagnosis), family history of cancer, prior attendance at a family cancer clinic, prior experience with genetic testing, and treatment history.	X		
**Perceived importance of genome sequencing** was measured using five items adapted from Hay et al. (2012), e.g., How important is it to you to learn about gene variants that may increase your chances of getting (another) cancer? Responses were scored on a Likert-scale from “not at all important” (1) to “very important” (5). Scores were averaged.Cronbach's alpha = 0.59	X		
**Knowledge of genomics** was measured using seven multiple choice study-developed questions assessing knowledge of the purpose of genome sequencing, likely frequency of informative results, cancers in which informative results are likely to be found, and utility of genome sequencing results. The number of correct responses was summed, with “Don't know” responses scored as incorrect, and an overall score (0–100%) calculated from the seven items.	X		
**Perceived ability to cope with results** was measured with four Likert-scale items adapted from Rosenberg et al. (2013), assessing perceived ability to cope with: a germline cancer gene variant result; a variant of uncertain significance; no cancer gene variants being found, and communicating germline results. E.g., I am confident I would be able to cope if a gene variant indicating that I and my family are at risk of some cancer, was found. Response options ranged from “strongly disagree” (1) to “strongly agree” (5). Scores were averaged across the four items, with higher scores indicating greater perceived ability to cope with results.Cronbach's alpha = 0.87	X		
**Intention to change behavior if a gene variant indicating cancer risk found** was measured with study developed Likert-scale items, e.g., If I knew I had inherited genes which increase my risk of cancer, I would be more careful with my diet. Response options ranged from “strongly disagree” (1) to “strongly agree” (5). Scores were averaged across the items, with higher scores indicating stronger intention to change behavior.Cronbach's alpha = 0.89	X		
**Fear of cancer recurrence**. The Concerns About Recurrence Questionnaire (CAR-Q) (Thewes et al., 2015) measured fear of cancer recurrence. Responses ranged from “none of the time/not at all” (0) to “all of the time/a great deal” (10). Scores were summed and averaged across the three questions, with higher scores indicating greater fear of cancer recurrence.Cronbach's alpha = 0.93	X	X	X
**Perceived susceptibility of (another) cancer** was measured with three items adapted from Kasparian et al. (2009). Participants self-rated their chances of developing another cancer from “much lower” (0) to “much higher” (4), and on a visual analogue scale from “no chance” (0%) to “will definitely” (100%). Participants also self-rated their chances of having a gene variant that increased their risk of cancer from “much lower” (0) to “much higher” (4). Likert-scale scores were multiplied by 25, then scores for all three items were averaged. Higher scores indicated greater perceived susceptibility of cancer.Cronbach's alpha = 0.66	X	X	X
**Attitude towards uncertainty:** The seven-item Attitude towards Uncertainty Scale (Braithwaite et al., 2002) measured attitude towards uncertainty in genome sequencing. Participants rated items on a Likert-scale from “strongly disagree” (1) to “strongly agree” (5). Scores were averaged across the items, with higher score indicating a negative attitude towards uncertainty.Cronbach's alpha = 0.87	X		
**Satisfaction with decision to have genome sequencing**. The six-item Satisfaction with Decision (SWD) scale (Holmes-Rovner et al., 1996) measured satisfaction with decision to have genome sequencing. Response options range from “strongly disagree” (1) to “strongly agree” (5). Scores were summed, with higher scores indicating greater satisfaction with decision.Cronbach's alpha = 0.92	X	X	X
**Genome sequencing related distress, positive experience and uncertainty**. The adapted Multidimensional Impact of Cancer Risk Assessment (MICRA) scale (Cella et al., 2002) measured distress, uncertainty and positive experiences specifically in the context of genome sequencing. Response options ranged from “never” (0) to “often” (5). The positive experience items were reverse coded, and item scores summed; thus, higher scores indicate greater distress, uncertainty, or positive experiences.			
Cronbach's alpha (distress) = 0.92 Cronbach's alpha (positive experience) = 0.88 Cronbach's alpha (family support) = 0.92 Cronbach's alpha (uncertainty) = 0.87		X	X
**Genome sequencing specific anxiety**. Impact of Events Scale (IES) (Horowitz et al., 1979; Thewes et al., 2001) measured genome sequencing specific anxiety. The scale measures the frequency of intrusions (unbidden thoughts, images, feelings) and avoidance (blunted sensation, behavioral inhibition, emotional numbness) Responses ranged from “not at all” (0) to “often” (5). A total score was obtained by summing the 15 Likert-scale items, with a higher score indicating greater anxiety about genome sequencing.Cronbach's alpha = 0.91		X	X
**Anxiety and depression**. The 14 item Hospital Anxiety and Depression Scale (HADS) (Zigmond and Snaith, 1983) measured general anxiety and depression. A total scale score was obtained by summing each item (range 0–42), with a higher score indicating greater anxiety and depression.Cronbach's alpha = 0.91		X	X
**Hope**. The 12 item Herth Hope Index (HHI) (Herth, 1992) measures hope and sense of meaning across three subscales: temporality and future, positive readiness and expectancy, and inter-connectedness. Responses range from “strongly disagree” (1) to “strongly agree” (4). Item scores are summed, with higher scores indicating greater hope.Cronbach's alpha = 0.88		X	X

Questionnaire data collection occurred from August 2016 to September 2020.

### Semi-Structured Interviews

Qualitative semi-structured telephone interviews were conducted by a trained qualitative researcher (NB). The interview guide (Supplementary Material—PiGeOn RisC T0 interview guide; Supplementary Material—PiGeOn RisC T1 interview guide; Supplementary Material—PiGeOn RisC T2 interview guide) development was informed by existing literature and input from a multidisciplinary advisory group, including consumers (i.e., cancer survivor trained in research processes). An initial draft was piloted with consumers. The interview questions relevant to this article addressed the types of uncertainty being experienced and strategies used to cope with uncertainty. Interview questions were iteratively modified over the course of the study. Interview participants were purposively sampled to ensure diversity in cancer type, age, and gender. Recruitment to the qualitative study continued until data saturation (no new themes emerging after three consecutive interviews) was reached at baseline (T0). All baseline (T0) interviewees were approached to participate in the 3 month (T1) follow up interviews, with additional participants approached to reach data saturation at this time point. All participants interviewed at baseline (T0) and 3 month (T1) follow up interviews were approached to participate in the 1 year (T2) follow up interviews. Interviewee demographic and disease characteristics were extracted from PiGeOn study baseline (T0) questionnaires. Interviews were conducted between August 2017 and October 2019.

### Analysis

Qualitative and quantitative data were collected concurrently and analysed separately. Data was then integrated using a matrix framed by the hypotheses to synthesise the quantitative and qualitative data (Bazeley, 2009).

#### Quantitative

Descriptive statistical analysis, correlations and regressions were conducted using IBM SPSS Statistics Version 26. Analysis of baseline (T0) data using Pearson's correlations was conducted to examine associations between perceived importance of genome sequencing, knowledge, perceived ability to cope with results, intention to change behavior if gene variant found, fear of cancer recurrence, perceived susceptibility of (another) cancer, satisfaction with decision to have genome sequencing, and attitude towards uncertainty. Cronbach alphas were conducted to determine reliability of scales used in this sample. Repeated measures ANOVA were conducted to investigate changes in means scores for measures administered at all three time points [fear of cancer recurrence, perceived susceptibility of (another) cancer, satisfaction with decision to have genome sequencing]. Paired sample *t-*tests were conducted to determine whether the mean difference for measures (perceived uncertainty about genome sequencing, genome sequencing related distress, genome sequencing related anxiety, general anxiety and depression, hope) administered at 3 (T1) and 12-months (T2) significantly differed. Analysis of longitudinal data using regressions was conducted to examine predictors of perceived uncertainty about genome sequencing and psychological outcomes—distress, fear of cancer recurrence, genome sequencing specific anxiety, general anxiety and depression, and hope. For each of these outcome variables (genome sequencing uncertainty, distress, fear of cancer recurrence, genome sequencing anxiety, general anxiety and depression, and hope), simple regressions with all potential predictor variables were conducted. All predictor variables that met the ≤ 0.20 significance threshold were then included in multiple linear regressions with each of the outcome variables.

#### Qualitative

Interviews were audio-recorded, transcribed verbatim, anonymized, uploaded to NVIVO 12 and subjected to recurrent cross-sectional (Grossoehme and Lipstein, 2016) thematic analysis (Braun and Clarke, 2006). A hybrid approach of thematic analysis was used, incorporating inductive (Boyatzis, 1998) and deductive (Crabtree and Miller, 1992) methods. The uncertainty coding framework developed from the baseline (T0) interview thematic analysis (Bartley et al., 2021) was applied to the 3 (T1) and 12-month (T2) interviews. Individual coding of an initial three transcripts was completed by two researchers (ZB, NB) to determine if the over-arching themes and sub-themes from the baseline (T0) coding framework was applicable. The framework was then applied to additional transcripts and further developed through an iterative process of review of subsequent transcripts. Once coded the data was organized into a time ordered matrix (Miles and Huberman, 1994; Grossoehme and Lipstein, 2016) to determine if themes and sub-themes differed across the time points. Relevant quotes to illustrate the identified themes were extracted. Differences in researcher interpretation of the data were resolved through discussion, with the multidisciplinary nature of the research team (psychology, bioethics, medicine) minimizing researcher bias regarding the meaning of the results (Berger, 2015).

## Results

### Quantitative Findings

Three hundred and fifty-three participants completed the baseline (T0) questionnaire (77% response rate), 346 participants completed the 3-month follow-up (T1) questionnaire (78% response rate), and 285 participants completed the 12-month follow-up (T2) questionnaire (70% response rate), with data for at least two time-points available for 359 participants. The majority (96%) of participants were interested in learning both cancer specific variants and secondary findings. Table 2 presents the demographic data of the majority female, English-speaking PiGeOn study participants. Most PiGeOn participants had a single primary diagnosis (70.2%), most commonly of a rare cancer (66.6%), and on average 8 years had passed since their diagnosis.

**Table 2:** Demographic and disease characteristics of the PiGeOn study participants.

**Variable**	***N =* 359 (%)**
**Sex**	
Female	239 (66.6)
Male	120 (33.4)
**Education**	
Don't know	1 (0.3)
Secondary school	75 (20.9)
Vocational training	54 (15.0)
University	229 (63.8)
**Occupation**	
Medical/science	30 (8.4)
Other	329 (91.6)
**Language spoken at home**	
English	293 (81.6)
Other	66 (18.4)
**Accessibility/Remoteness Index of Australia^a^**	
Major city	294 (81.9)
Inner regional	42 (11.7)
Outer regional	16 (4.5)
Remote	3 (0.8)
Unknown/overseas	4 (1.1)
**Visited a Family Cancer Clinic^a^**	
Don't know	15 (4.2)
Yes	103 (28.9)
No	239 (66.9)
**Marital status**	
Single	93 (25.9)
Married/living with a partner	243 (67.7)
Separated/divorced	16 (4.5)
Widowed	7 (1.9)
**Parental status^a^**	
Children	195 (54.8)
No children	161 (45.2)
**Multiple primary diagnosis**	
Yes	107 (29.8)
No	252 (70.2)
**Cancer incidence**	
Rare	239 (66.6)
Less common	23 (6.4)
Common	97 (27.0)
**Age at consent (years)**	
Mean (SD)	43.31 (13.98)
Range	16–83
Median (IQR)	39.0 (17)
**Socio-Economic Indexes for Australia**	
Mean (SD)	7.41 (2.61)
Range	1–10
**Time since first diagnosis (years)**	
Mean (SD)	8.06 (9.71)
Range	0–52.17
Median (IQR)	4.17 (8.75)

a *participant numbers do not add up to 359 due to missing data; percentages reported are valid percent's*.

Participants had on average negative attitudes towards uncertainty (*M* = 4.03, SD 0.68) at baseline (T0, Table 3). Perceptions of uncertainty about genome sequencing was low at both 3 months (T1, *M* = 8.23, SD 7.37) and 12 months (T2, *M* = 7.95, SD 7.64) follow up, and did not change significantly over time [*t*(210) = 0.660, *p* = 0.510]. At baseline (T0), participants had high perceived importance of genome sequencing, moderate knowledge of genomics, high perceived ability to cope with genome sequencing results, and high intention to change behavior if a gene variant indicating cancer risk was found. Mean fear of cancer recurrence scores were above the clinical cut-off of ≥10 for this scale (Thewes et al., 2015) at all time-points. Participants had low levels of anxiety and depressive symptoms at over the 12 months, with mean scores on genome sequencing anxiety (Horowitz et al., 1979; Thewes et al., 2001), and general anxiety and depression (Zigmond and Snaith, 1983) below the clinical cut-offs for these scales, at all time-points. Participants had high levels of hope across the 12 months, and low to moderate levels of genome sequencing related distress.

**Table 3:** PiGeOn participants' knowledge, attitude, and psychological outcomes over 12 months.

**Variable**	**Baseline (T0) (*n =* 353)**	**3- month (T1) (*n =* 346)**	**12-month (T2) (*n =* 285)**	**Significance test, *p-*value**
**Perceived importance of genome sequencing**				
Mean (SD)	3.76 (0.55)			
Range	1.4–5.0			
**Knowledge of genomics**				
Mean (SD)	0.47 (0.24)			
Range	0–1			
**Perceived ability to cope with genome sequencing results**				
Mean (SD)	4.17 (0.67)			
Range	1–5			
**Intention to change behavior if gene variant indicating cancer risk was found**				
Mean (SD)	4.25 (0.66)			
Range	1–5			
**Attitude towards uncertainty**				
Mean (SD)	4.03 (0.68)			
Range	1.57–5			
**Fear of cancer recurrence**				
Mean (SD)	13.76 (815)	12.36 (7.94)	11.89 (8.15)	*F*_(1.90, 494.21)_=10.26, *p* = 0.000
Range	0–30	0–30	0–30	
**Perceived susceptibility of (another) cancer**				
Mean (SD)	64.78 (16.44)	65.09 (16.33)	65.87 (17.82)	*F*_(1.94, 503.19)_ = 0.69, *p* = 0.500
**Satisfaction with decision to have genome sequencing**				
Mean (SD)	26.17 (3.17)	26.74 (3.89)	26.33 (4.13)	*F*_(1.93, 494.50)_=2.33, *p* = 0.100
**Genome sequencing anxiety**				
Mean (SD)		5.78 (9.94)	6.38 (11.82)	*t*(265) = −1.160, *p* = 0.247
**General anxiety and depressive symptoms**				
Mean (SD)		7.98 (6.66)	8.53 (7.30)	*t*(265)=-1.610, *p* = 0.109
**Anxiety subscale**				
Mean (SD)		5.15 (4.09)	5.48 (4.44)	*t*(265)= −1.617, *p* = 0.107
**Depression subscale**				
Mean (SD)		2.83 (3.25)	3.04 (3.45)	*t*(265) = −1.205, *p* = 0.229
**Hope**				
Mean (SD)		39.30 (5.52)	39.23 (6.07)	*t*(211) = 0.203, *p* = 0.839
**Distress**				
Mean (SD)		22.34 (9.49)	23.20 (10.29)	*t*(210) = −1.354, *p* = 0.177
**Perceived uncertainty about genome sequencing**				
Mean (SD)		8.23 (7.37)	7.95 (7.64)	*t*(210) = 0.660, *p*=0.510

**Figure 1:**
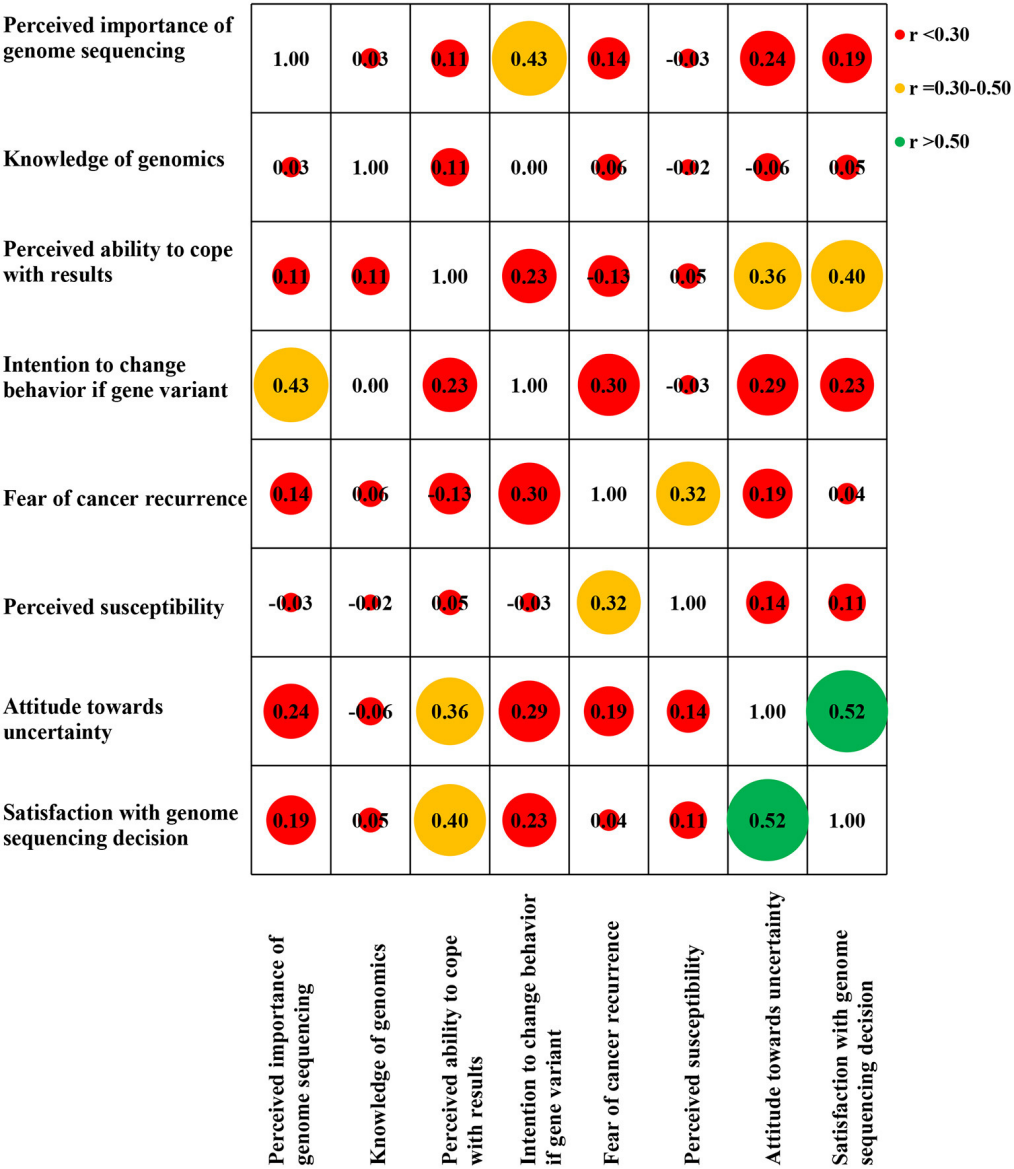
Baseline (T0) variables correlation matrix heat map (Pearson correlation coefficient).

We found weak correlations between negative attitudes toward uncertainty and greater perceived importance of genome sequencing [*r*(350) = 0.24, *p* < *0*.01], intention to change behavior if a gene variant indicating cancer risk was found [*r*(349) = 0.29, *p* < *0*.01], fear of cancer recurrence [*r*(348) = 0.19, *p* < *0*.01], and perceived susceptibility for (another) cancer [*r*(348) = 0.14, *p* < *0*.01] at baseline (T0). Greater perceived ability to cope with results [*r*(349) = 0.36, *p* < *0*.01] was moderately correlated with negative attitudes towards uncertainty. Greater satisfaction with decision to have genome sequencing [*r*(350) = 0.52, *p* < *0*.01] was strongly correlated with negative attitudes towards uncertainty (Figure 1).

As there was no significant change in perceived uncertainty about genome sequencing or the psychological outcomes (with the exception of fear of cancer recurrence) over time, we report only on 12-month psychological outcomes in this article.

**Figure 2:**
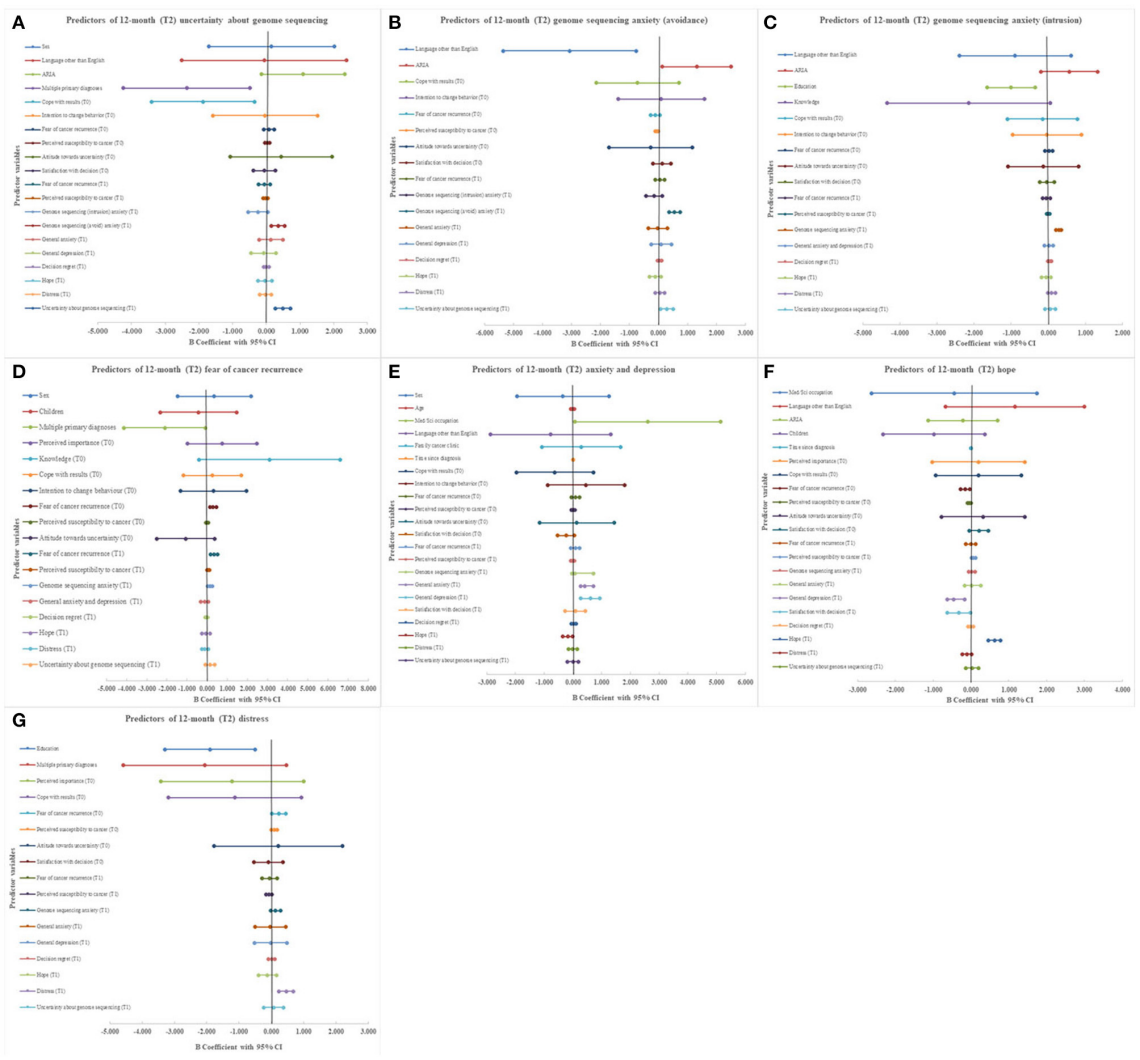
Forest plots presenting results of multiple regression analysis for predictors of psychological outcomes in cancer patients undergoing genome sequencing. **(A)** 12 month (T2) uncertainty about genome sequencing. **(B)** 12 month (T2) genome sequencing anxiety (avoidance). **(C)** 12 month (T2) genome sequencing anxiety (intrusion). **(D)** 12 month (T2) fear of cancer recurrence. **(E)** 12 month (T2) anxiety and depression. **(F)** 12 month (T2) hope. **(G)** 12 month (T2) distress.

Multiple primary cancer diagnoses [B = −2.364 [−4.238, −0.491], *p* = 0.014], baseline (T0) lower perceived ability to cope with results [B = −0.1.881 [−3.403, −0.359], *p* = 0.016], greater anxiety (avoidance subscale) about genome sequencing [B = 0.347 [0.148, 0.546], *p* = 0.0012] at 3 months (T1), and greater perceived uncertainty about genome sequencing [B = 0.494 [0.267, 0.721] *p* = 0.000] at 3 months (T1) significantly predicted greater perceived uncertainty about genome sequencing at 12 months (T2, Figure 2A). Greater perceived uncertainty about genome sequencing at 3 months (T1) significantly predicted greater anxiety (avoidance subscale) about genome sequencing at 12 months (T2, B = 0.291 [0.072, 0.509], *p* = 0.009) (Figure 2B). Attitude towards uncertainty at baseline (T0) did not significantly predict psychological outcomes (Figures 2A–G).

### Qualitative Findings

Semi-structured interviews [*N* = 20 at baseline (T0), *N* = 23 at 3 months (T1), and *N* = 24 at 12 months (T2)] revealed that, while participants were motivated to pursue genome sequencing as a strategy to reduce illness uncertainty, genome sequencing generated additional practical, scientific and personal uncertainties (Han et al., 2017). At baseline (T0), four themes emerged from the qualitative data: 1. Genome sequencing to reduce illness uncertainty; 2. Genome sequencing to reduce uncertainties about relatives' risk; 3: Uncertainty generated by genome sequencing; and 4. Resilience and coping with uncertainty (Bartley et al., 2021). Some uncertainties were consistently present over the 12 months, while others dropped off or emerged over time (Table 4).

**Table 4:** Uncertainty themes and sub-themes discussed by PiGeOn interviewees over 12 months.

**Themes and sub-themes**	**Baseline (T0)**	**3-month (T1)**	**12-month (T2)**
**Genome sequencing to reduce illness uncertainty**			
Etiology	X	X	X
Own illness risk	X	X	X
Disease trajectory uncertainty		X	
Previous genetic testing		X	X
Illness uncertainty most salient	X	X	X
Continued uncertainty over bad news		X	X
**Genome sequencing to reduce uncertainty about relatives' risk**			
Relatives illness risk	X	X	X
Children	X	X	X
Family planning	X	X	
Family history of illness		X	
Potential impact on relatives	X	X	X
Family communication	X	X	X
Family history influence on communication			X
**Uncertainty generated by genome sequencing**			
No uncertainty	X	X	X
Practical—uncertain knowledge	X	X	X
Practical—uncertain of study processes		X	X
Practical—insurance/discrimination	X	X	X
Scientific—limitations of science	X	X	X
Scientific—result ambiguity	X	X	
Scientific—likelihood of results			X
Personal—emotional reaction to results	X	X	X
Personal—life choices	X	X	X
Personal—ethical uncertainty		X	
Decisional uncertainty (to have/not have; which results if any)	X	X	X
Uncertainty reduces/become episodic over time		X	X
**Resilience and coping with uncertainty**			
Resilience	X	X	X
Mobilizing coping—information seeking	X	X	X
Mobilizing coping—lifestyle	X		X
Mobilizing coping—plan of action	X		
Affect coping—seeking professional support	X		X
Affect coping—seeking informal support	X	X	
Affect coping—positive attitude	X	X	X
Affect coping—trust in experts/research		X	X
Affect coping—reduce expectation			X
Affect coping—don't worry about things outside your control		X	X
Buffering coping—acceptance	X	X	X
Buffering coping—avoidance	X	X	X
Buffering coping—live in the now	X	X	
Unhealthy behaviors	X		
Worry/anxiety	X	X	X

#### Genome Sequencing to Reduce Illness Uncertainty

Participants viewed genome sequencing as a way to reduce their illness-related uncertainty throughout the 12 months. They felt that genome sequencing would increase their understanding of the origin of their cancer and provide clearer estimates of their risk of developing another cancer. At 3 months (T1), four participants discussed disease trajectory uncertainty and that genome sequencing results have the potential to reduce this in that it may give them access to new and more targeted treatments.

*What I'm hoping is that at some stage there will be some more targeted immunotherapy or more personalized therapy which is developed. I'm just hopeful that the research gets spurred on really quickly and that if I ever really do need to call upon more treatment that the genome sequencing might have made me suitable for some new up-and -coming treatments (Female, 37 years, gastrointestinal tract cancer)*.

For some participants, adding to this motivation was previous experience with uninformative genetic testing and the hope that genome sequencing would provide answers.

Concerns about genome sequencing were secondary to disease-related uncertainties. At the 3 (T1) and 12 months' (T2) follow-up interviews, most participants placed even more importance on reducing illness-related uncertainty, noting that even bad news was preferable to not knowing.

*At the end of the day, I get more concerned with my scans than the gene sequencing and, I guess, that's in the back of my mind (Female, 37 years, central nervous system cancer)*.

*I mean, nobody wants bad news but if you know something, you might be able to do something… if you find something now, well it's better than not knowing (Male, 52 years, parathyroid cancer)*.

However, during the follow-up interviews, a few participants did express a desire to remain uncertain over receiving bad news.

*I'd rather just be completely oblivious for now… I think it would be finding something that they know nothing about, or something that I can't do anything about. Like, if I was going to get dementia at this point in time, if you get it you get it. I'd probably rather be blissfully unaware (Female, 27 years, ovarian cancer)*.

### Genome Sequencing to Reduce Uncertainties About Relatives' Risk

Similarly, over the 12 months participants continued to view genome sequencing as a tool to reduce their uncertainty about their relatives' risk of cancer and were still uncertain about how relatives would react to results, or which results they would communicate to relatives and/or how they would approach that communication. At 3-month (T1) follow-up interviews, participants discussed how a family history of illness added to their motivation to have genome sequencing to reduce uncertainty about relatives' risk.

*My mum died of cancer three years ago, my brother has had a cancer six years ago… another brother had a benign cancer. So, it is something that is in our family and I would be glad if there is something in my genetic code that can help the rest of the family, particularly the kids… I have three brothers… The fourth one is the youngest and he's 35, so far, he hasn't had any problem but he's the one that obviously is most interested in understanding if there is a genetic explanation. I would be, not happy*—*but I'd say curious to know if there is something genetic. Because it is kind of strange that in same family, out of six people, four had a cancer. If it's not genetic, that means that either we are very unlucky or there was something in our family history that was not correct (Male, 44 years, central nervous system cancer)*.

At 12-month (T2) follow-up, interviewees discussed how a family history of cancer had facilitated communication with their family members about undergoing genome sequencing. However, this family history also contributed to their feelings of uncertainty around how family members would react to genome sequencing results.

*Mum's a little bit apprehensive, she's like, “Well, do you really think you need to know?” And it's like, “Well, yeah. I think it's good.” I think she's just, she's got my sister and myself are the only children they've got, and both of their kids had cancer. So, from a parent's perspective you don't want to think your kids ever are going to get sick again. So, I suppose for her, she would find it hard to be given that information there and then (Female, 42 years, blood cancer)*.

#### Uncertainty Generated by Genome Sequencing

Participants continued to experience genome sequencing specific uncertainties throughout the 12 months. This included scientific uncertainties, such as recognizing the limitations of science; personal uncertainties, such as potential emotional and behavioral reactions to results; and practical uncertainties, such as lack of genomic knowledge. At follow-up interviews a new practical uncertainty emerged involved being unsure about study processes, for example, where their sequencing was up to and when they would be receiving results. Additionally, a new scientific uncertainty about the likelihood of a gene variant indicating risk being found through the genome sequencing emerged, while scientific uncertainty related to result ambiguity was no longer discussed by interviewees at 12-months (T2). Two participants discussed ethical uncertainty in their 3-month (T1) interview, being unsure if genomics is *playing god a little bit*, and whether patients should access genome sequencing when there is still a level of scientific uncertainty, especially when cancer patients are emotionally vulnerable.

*I wonder how it's all going, cause I hadn't heard anything, so yeah, sort of apprehensive wondering, how it's all going, if they found anything or things like that (Male, 56 years, genitourinary cancer)*.*I think before I was very certain, probably something would… oh, I don't know, I'm not so sure. [Doctor] said as well, a lot of it is probably autoimmune-related so I don't know if anything can really be shown through the study… I think I was more positive before but now I'm, like, nothing's ever found (Female, 31 years, head and neck cancer & sarcoma)*.

*A lot of people put a lot of emphasis needing to send samples off overseas and paying out-of-pocket expenses for it and trying to get samples moved between facilities because they're hunting for that magic bullet. We're still in a stage where there's a lot of hype and a lot of promise and not actually a lot of effect, so that worries me and I think that leads itself to being exploited by a whole bunch of practitioners and organizations that will trade on the hope that DNA therapeutics will give people and predictive testing will give people, but the science just isn't there (Male, 35 years, gastrointestinal tract cancer)*.

At three (T1) and 12 months (T2), many interviewees discussed their genome sequencing related uncertainty having reduced over time or having become episodic. Interviewees were not thinking about these uncertainties constantly over the 12 months but were prompted by study processes such as questionnaires or the realization that they would be receiving results soon.

*I suppose I thought I would get the results, so I was trying to brace myself with what would happen. Which is the first time in the whole year that I've done that. So that was interesting for me to be a little bit uptight at times… and hubby would say, “What's wrong?” and I'd go, “Oh, it's just, I suppose I'm going to get these results and it's just going to be interesting, what my life is going to be like once I get that information” (Female, 42 years, blood cancer)*.

#### Resilience and Coping With Uncertainty

Resilience to uncertainty was present across the 12 months. Over the 12 months, interviewees continued to engage in buffering coping strategies, such as acceptance and avoidance, but no longer discussed living in the now as a coping strategy for uncertainty at 12 months (T2). Interviewees continued to engage in mobilizing coping strategies such as information-seeking and maintaining a healthy lifestyle over the 12 months, while having a plan of action as a coping strategy for managing uncertainty was only raised by interviewees at baseline (T0). Interviewees continued to use affect focused coping strategies, such as seeking professional and informal support and maintaining a positive attitude across the 12 months. Interviewees mentioned additional affect coping strategies such as trusting in the experts and research, reducing their expectations of genome sequencing, and not worrying about things outside of their control in their follow-up interviews.

*Just sort of left it in the hands of the scientists (Female, 41 years, breast cancer)*.

*If you have no expectations then you can't be disappointed, you know. So, bottom line is I really don't have any expectation… the more expectation you put on things, the more chance you are going to worry or be disappointed if the outcome's not what you want (Male, 47 years, gastrointestinal tract cancer & granular cell tumor)*.

*I don't really worry about things I can't control, so I haven't thought about it, because I'll either get results or I won't. It's one thing I learnt during cancer, it's really unhelpful to worry about things you can't influence, and it's very important to know the difference (Female, 43 years, blood cancer)*.

## Discussion

The results of this study provide further support for the published literature on the topic of cancer patient perceptions of uncertainties related to genomics. The findings indicate that patients are motivated to undergo genome sequencing as a strategy to reduce their illness-related uncertainty, and that genome sequencing generates practical, scientific, and personal uncertainties for patients reinforce those of previous studies (Bartley et al., 2020b). Additionally, the findings build on the existing knowledge by providing important information about patients' experience of uncertainties while waiting for results, as well as the factors contributing to ongoing uncertainties and the psychological impact of the uncertainties.

Previous research has found that individuals without a history of cancer who have negative attitudes towards uncertainty are more likely to undergo predictive genetic testing for colon and breast cancer (Braithwaite et al., 2002), and cancer patients are motivated to pursue genomic testing by a desire to reduce illness uncertainty (Claes et al., 2004; Sanderson and Wardle, 2008; Bartley et al., 2020a). Mishel's (1990) reconceptualization of the Uncertainty in Illness Theory, proposes that patients experiencing ongoing uncertainty may use coping strategies to adapt to their uncertainty. Our participants who had agreed to genome sequencing, generally had more negative attitudes towards uncertainty related to genome sequencing at baseline (T0), and continually discussed a desire to reduce illness related uncertainty by gaining genomic information, across the 12 months. Our participants however reported low levels of perceived uncertainty associated with genome sequencing results and future plans at 3 (T1) and 12-month (T2) follow-up, suggesting that cancer patients undergoing genome sequencing adapt to their perceived uncertainties over time. Our qualitative data provide further support for this quantitative finding, in that while patients discussed a variety of scientific, personal and practical uncertainties while waiting for their results, they also discussed their uncertainty either reducing or becoming more episodic over the 12-month period. Our participants considered themselves quite resilient to uncertainty and engaged in a variety of coping strategies to help them deal with their uncertainty. This resilience and coping strategies could explain why even though our participants held more negative attitudes towards uncertainty at baseline, their perceived uncertainty about genome sequencing was low at follow up, as they were effectively coping with their uncertainty.

Uncertainty is inherent in cancer. Uncertainty about prognosis, treatment options and effectiveness can produce feelings of uncontrollability, and has been associated with anxiety in cancer patients (Tan et al., 2016; Curran et al., 2017). While we did not find a relationship between attitude towards uncertainty at baseline and perceived uncertainty about genome sequencing or psychological outcomes at 12 months, our results did show an inter-relationship between perceived uncertainty about genome sequencing and anxiety specifically related to genome sequencing. Greater anxiety (avoidance subscale) about genome sequencing significantly predicted greater perceived uncertainty at 12 months (T2) and greater perceived uncertainty about genome sequencing at 3 months (T1) significantly predicted greater anxiety (avoidance subscale) about genome sequencing at 12 months (T2). This relationship is not surprising as uncertainty reduces how efficiently and effectively individuals can prepare for the future and is a basic cognitive process that contributes to anxiety (Grupe and Nitschke, 2013; Carleton, 2016). Participants experiencing ongoing uncertainty about their genome sequencing may not be able to adequately prepare themselves for receiving their results and therefore experience anxiety about the genome sequencing. This is supported by the finding that lower perceived ability to cope with results at baseline (T0) was also a predictor of greater perceived uncertainty about genome sequencing at 12 months (T2). Further supporting this was the acknowledgment by participants across the 12 months that they were uncertain about how they would emotionally and/or behaviorally cope with their genome sequencing results.

Tercyak et al. (2001) found that women participating in genetic counseling and testing for breast and ovarian cancer risk experienced anxiety while waiting for their results. Specifically, women who were high monitors (closely attending to threat-relevant cues) were more likely to experience anxiety when confronted with the uncertainty involved with waiting for cancer risk information. Lazarus and Folkman's (1984) Transactional Model of Stress and Coping proposes that coping strategies are implemented based on how individuals appraise the threat to their well-being. This appraisal is based on the threats personal significance and the individuals perceived ability to cope with outcome. Coping strategies are then engaged based on the appraisal. For example, affect coping strategies such as seeking professional and informal support and maintaining a positive attitude are likely to be engaged when the uncertainty is appraised as out of the patients control. The literature suggests that coping strategies are most effective when the strategy matches the controllability of the threat (Gooding et al., 2006), and that accepting uncertainty is likely to be associated with more helpful coping (Mishel, 1990; Carleton, 2016; Curran et al., 2017). This study also found that coping styles of participants may be an important predictor of psychological outcomes. Our study found that uncertainty about genome sequencing while awaiting results is episodic and that some coping strategies may be engaged over time, while others may be more specific to certain time points. For example, having a plan of action based on the worst-case scenario may be a coping strategy that helps patients deal with the uncertainty associated with how they will respond to results, which facilitates the patient's decision making to have genome sequencing. Whereas, coping strategies to manage affect, such as seeking professional support, maintaining a positive attitude and trusting in the experts or research may be useful while waiting for results, as the decision to have genome sequencing is made and no longer in their control. Understanding which coping strategies are most useful throughout the genome sequencing process could help patients manage their uncertainty, and potentially ameliorate psychological outcomes such as anxiety.

Increasing our understanding of uncertainties that patients experience while waiting for genome sequencing results provides patients and healthcare professionals with useful information about the sources and types of uncertainties which may emerge throughout the process. Healthcare professionals could utilize this information to discuss potential uncertainties with patients during their decision making to pursue genome sequencing, promoting informed consent and realistic expectations of this clinical tool (Biesecker et al., 2014; Han et al., 2017). Healthcare professionals can support patients by coaching them to understand that not all uncertainty needs to be resolved (Newson et al., 2016) and helping them to reappraise some uncertainties as opportunities rather than threats (Mishel, 1990). Knowing about the patient experience can help healthcare professionals provide anticipatory guidance to participants and help them to manage the uncertainties that may be construed as a further threat to their cancer prognosis. Specifically, healthcare professionals can help patients to identify and engage in effective coping strategies to manage their uncertainties while waiting for results, which in turn could reduce their likelihood of developing further anxiety about outcomes from genome sequencing.

Given the diverse range of uncertainty related to genome sequencing revealed in this study and others, we believe that measures of uncertainty need careful consideration. Developed to measure attitude towards uncertainty related to medical tests, we believe that the Attitude towards Uncertainty scale does not capture the complexity of uncertainty experienced by patients undergoing genome sequencing. Since designing this study, a more relevant tool for capturing patient-perceived uncertainties associated with genome sequencing has been developed, the Perceptions of Uncertainties in Genome Sequencing (Biesecker et al., 2017), which we believe will be of use to researchers investigating uncertainties in genome sequencing.

### Limitations and Strengths

The results of the current study are limited by sampling bias, including overrepresentation of female participants, Caucasian participants, highly educated participants, and participants with rare cancers, which limits the generalizability of our findings.

While reporting on the ongoing uncertainty experienced by patients waiting for genome sequencing results is an important addition to the literature in this area, all data were collected prior to participants receiving their genome sequencing results. We believe that further studies would be strengthened by collecting uncertainty data post-results as well, to investigate the impact which different results have on participant uncertainty. Nevertheless, methodological strengths of this study were its longitudinal mixed-methods design. Following our participants over the 12-month period while waiting for genome sequencing results allowed us to investigate changes in uncertainty over time, as well as the impact of uncertainty on psychological outcomes. Utilizing qualitative and quantitative methods allowed us to both investigate relationships between variables, and also describe experiences of participants.

### Implications and Future Research

This study demonstrated the complexity of uncertainty generated by genome sequencing for cancer patients and provides further support that while patients are motivated to pursue genome sequencing to reduce illness uncertainty (for self and relatives) it also generates uncertainties (Bartley et al., 2020b). Additionally, the results suggest that uncertainty while waiting for genome sequencing is low, but that patients with uncertainty related to genome sequencing may also be vulnerable to genome sequencing related anxiety. Understanding how uncertainty coping strategies may help patients adapt to their uncertainty over time could assist health care professionals manage patient uncertainty, which in turn may ameliorate psychological outcomes such as anxiety.

## Data Availability Statement

The raw data supporting the conclusions of this article will be made available by the authors, without undue reservation.

## Ethics Statement

The studies involving human participants were reviewed and approved by The St Vincent's Hospital Human Research Ethics Committee (HREC/16/SVH/24). The patients/participants provided their written informed consent to participate in this study.

## Author Contributions

PB, MBe, MBa, TS, BB, and NB contributed substantially to the conception and design of the study. NB and CN contributed substantially to data acquisition. NB, ZB, CN, TS, and PB contributed substantially to the analysis and interpretation of data. NB drafted the manuscript. BB, MBe, MBa, ZB, CN, TS, and PB revised the manuscript. All authors had full access to all the data in the study, gave final approval of the current draft, and agree to be accountable for all aspects of the work.

## Conflict of Interest

The authors declare that the research was conducted in the absence of any commercial or financial relationships that could be construed as a potential conflict of interest.
