# Supplementary material for: Cancer Patient Experience of Uncertainty While Waiting for Genome Sequencing Results
Source: Front Psychol. 2021 Apr 22;12:647502. doi: 10.3389/fpsyg.2021.647502 (PMC8100530; doi:10.3389/fpsyg.2021.647502)
Supplement: Supplementary file 1 [file Data_Sheet_1.PDF]

ST VINCENT'S HOSPITAL SYDNEY

A FACILITY OF ST VINCENT'S HEALTH AUSTRALIA

Genetic Cancer Risk in the Young Study

Short title: Cancer Risk Study

Participant Information Sheet/Consent Form

St Vincent's Hospital Sydney

The study is being conducted by the following investigators

**Principal Investigator:**	Professor David Thomas
**Other investigators:**	Professor Allan Spigelman
	Dr Mandy Ballinger

1. Introduction

You are invited to take part in this research project. This is because you or a family member have had cancer. You will be asked to donate a sample of blood which will be used for genetic research.

This Participant Information Sheet/Consent Form tells you about the research project. It explains the tests and treatments involved. Knowing what is involved will help you decide if you want to take part in the research.

Please read this information carefully. Ask questions about anything that you don't understand or want to know more about. Before deciding whether or not to take part, you might want to talk about it with a relative, friend or your local doctor.

Participation in this research is voluntary. If you don't wish to take part, you don't have to. You will receive the best possible care whether or not you take part.

If you decide you want to take part in the research project, you will be asked to sign the consent section. By signing it you are telling us that you:

- Understand what you have read
- Consent to take part in the research project
- Consent to have the tests and treatments that are described
- Consent to the use of your personal and health information as described.

You will be given a copy of this Participant Information and Consent Form to keep.

Before you decide whether or not you wish to participate in this study, it is important for you to understand why the research is being done and what it will involve. Please take the time to read the following information carefully and discuss it with others if you wish.

2. What is genetic research?

Genes are made of DNA – the chemical structure carrying your genetic information that determines many human characteristics such as the colour of your eyes or hair. Researchers study genes in order to understand why some people have a certain condition such as cancer and why some people do not. Our genes are like a set of instructions that help bodies to work

properly. We all have approximately 20 000 genes. This is called our genome. Some of our genome is unique to each of us, but we do share most of it with our relatives.

Cancer is caused by alterations (variants) in our genes that make cells grow out of control and spread abnormally. Variation in our genomes is normal. Some variants can increase a risk of developing certain diseases (such as cancer), while others (such as variants that determine eye colour) do not. Some, but not all, genetic variants that increase the risk of developing cancer are inherited (passed on from parent to child) and have implications for all blood relatives. We already know about some of these, but there is still much more to learn.

Whole genome sequencing
Until now we have only been able to look for inherited variants in a few genes. For example we know that breast cancer is sometimes caused by a variant in the BRCA1 or BRCA2 gene. Recent developments in science and technology mean that it has become possible to look at the whole of a person's genome at once, to try to understand more about the genetic causes for disease. In this study we are planning to see whether there are any gene variants in all 20 000 of your genes, using a technique called whole genome sequencing.

Whole genome sequencing involves obtaining the sequence of all of your 20 000 genes in one test. When your sequence is examined, results may show that a gene is normal, or show a variant thought to cause disease, or a variant that has unknown significance. Some of this information might be relevant to your or your family's risk of developing cancer, but there is a small chance that whole genome sequencing may detect a variant in your genome that is not related to your risk of cancer. This is called an 'incidental finding'. It's also important to remember that if nothing is found, this does not rule out a genetic contribution to your cancer.

In this study, we plan to offer you information about your risk of cancer and information about some other conditions if we are sure about the medical significance of the finding. We do not plan to offer you information if it is of uncertain significance. It is up to you to decide how much of your information you would like to be told. Some people like to know this information, while others prefer not to be told. It may help to think in advance about what you might do with any information you may find out.

3. What is the purpose of this research?
The aim of the study is to understand more about the genetic variants that contribute to inherited cancer. This is important for people with cancer and their families. We hope that this knowledge will lead to more personalised cancer treatments with better outcomes, improved cancer screening, increased options for reducing cancer risks and more fully informed lifestyle and reproductive decisions. We aim to also assess health related costs.

In this study we would also like to understand what people think and feel about this type of genetic testing.

4. What does participation in this research involve?

- *Completing the consent form*

  Study staff will go through the consent form and answer any questions you may have. If you decide to participate, we will ask you to sign the consent form before taking any part in the study. Take your time with considering this information.

- *Completing the initial questionnaire*

  We will ask you some questions about your health, medical history, lifestyle and family medical history as well as your attitudes towards and knowledge about genetic testing. This survey will take about 30 minutes and may be face to face or over the telephone. We may ask you similar questions in the future. You do not have to answer these questions if you prefer not to.

- *Completing the follow up questionnaires*

We will contact you by mail or telephone with some follow up questions 3 months and 12 months after you agree to take part in the study. The follow up questions will take about 30 minutes to answer.

- *Providing a blood sample*

We will arrange for you to give a blood sample for the study. If you are a current patient, the blood sample will be taken at one of your treating centres on a routine visit. If you are a previous patient or a relative of a patient, we will give you written instructions as to how to arrange this at your local pathology service or GP, at no cost to yourself.

- *Allowing the study to obtain a small part of any cancer tissue that has been removed in the past or may be removed in the future.*

We will request the tissue directly from the pathology service. You can be reassured that any tissue used for the study will not interfere with your diagnosis or treatment and we will not ask you to have an additional tissue sample taken for the study.

- *Allowing medical information about yourself to be collected from cancer registries, hospital records, medical rooms and the Australian Institute of Health and Welfare.*

We will access and link data from sources containing information about your health, medication and treatment including cancer registry records.

- *Agreeing to ask relevant family members if they are interested in being invited to participate in the study.*

We will not reveal your personal details to other family members.

- *Participating in an interview*

We may ask you to participate in 3 interviews. These will occur around the time you consent to the study and 3 and 12 months later. These may be face to face or over the telephone and will take around 30 minutes each time. We will ask you how you feel about whole genome sequencing generally.

- *Considering optional components of the study*

We will ask you to consider being involved in some additional research components. This will involve additional separate consent forms. We will ask your permission to access your Medicare and Pharmaceutical Benefits Scheme information. You can still take part in the rest of this study even if you do not want us to access your Medicare and PBS details. If you choose to consent to the study accessing your Medicare and PBS data, at the completion of the study we will ensure that all confidential information contained in hard copy will be destroyed using shredders and disposed of in confidential waste bins. Electronic files containing confidential information will be deleted from all systems.

We will also ask your permission to use your re-identifiable data and specimens in future unspecified research projects. You many choose not to give permission for any component of the study.

Participation in this study will not cost you anything and you will not receive any payment for your involvement in the research. If a gene variation that is significant to your health is detected and you choose to have confirmatory testing in a clinical laboratory, there may be costs associated with this that you will have to cover.

5. Other relevant information about the research project

There will be approximately 1000 families participating in this study from New South Wales. Several hospitals/clinics will be involved including St Vincent's Hospital, Chris O'Brien Lifehouse, the Hereditary Cancer Clinic at Prince of Wales Hospital and the Familial Cancer Service at Westmead Hospital.

6. Do I have to take part in this research project?
Participation in any research project is voluntary. If you do not wish to take part, you do not have to. If you decide to take part and later change your mind, you are free to withdraw from the project at any stage.

If you do decide to take part, you will be given this Participant Information and Consent Form to sign and you will be given a copy to keep.

Your decision whether to take part or not to take part, or to take part and then withdraw, will not affect your routine treatment, your relationship with those treating you or your relationship with St Vincent's Hospital Sydney.

7. What are the possible benefits of taking part?
You will be contacted if the testing shows important information about you, and you will be asked if you wish to know the results. The results may be important to you as they may provide:
- Information about risk of an inherited condition
- Information that might influence a decision to have children
- Information that might affect your ability to obtain insurance or employment.

In addition, if the testing shows important information about your relatives contact with your relatives about the testing is encouraged. You may wish to do this yourself or ask the researchers to contact them on your behalf.

Should you inform your relatives and they wish to know your results, genetic counselling will be arranged by the Study co-ordinator to explain what the results mean for you and to support you as necessary.

It will be necessary to refer you for re-testing by genetic services outside this research project. There may be some costs associated with re-testing that will not be covered by the study and will have to be covered by you, if you choose to go ahead.

8. What are the possible risks and disadvantages of taking part?
Genetic testing involves the study of genetic material (typically DNA) that is shared with your blood relatives. Genetic research is undertaken for many reasons, including discovering more accurate ways of predicting disease within a group of people, or in people where there is strong family history or predisposition of disease.

Genetic testing may raise important issues. Although few may be expected to arise, your awareness of this is important for you to think about and carefully consider before agreeing to participate. Genetic information may have implications for you and your blood relatives.

Learning about the results from genetic research might affect you and your family emotionally. In some cases, the result may give certainty that you do not have a disease but could also create uncertainty or be upsetting; if for instance, the test indicates an increased risk of developing a disease which has no known prevention, treatment or cure.

It is important to understand that results from genetic research will usually not indicate that you have a disease or disorder, or whether you will develop it. Research may only show that you have an increased **risk** of developing a disease or disorder. Even then, there is no guarantee that you will develop the condition or any indication of the likely age you might get the disease or how serious the disease might be.

You may learn information from your test result about inherited diseases or disorders that may affect others, such as your brothers or sisters. This could interfere with family relationships. You may be faced with the decision to make the family aware of the existence of genetic information. Family members may or may not wish to know this information.

Any research results that could be of significance to you or your family will need to have the tests repeated and the results verified. This will involve having a blood sample taken and having it retested in an accredited testing laboratory. This is standard practice for all patients receiving the results of genetic testing. There may be some charges to you. Counselling may be provided free of charge if it is appropriate. Before a test is repeated to verify a research finding, you will be informed about the possible risks involved for you. This is especially important for individuals who are found to have a genetic mutation that is associated with an increased risk of developing a disease such as cancer or heart disease.

- **You may have a cancer gene variant**

There is a chance that you have a gene variant that makes you more likely to develop cancer. Other members of your immediate family and other blood relatives may also have the same gene variant. This gene variant could be passed on to the next generation. If you do have a gene variant, you and possibly other family members may have a higher risk of developing cancer, but we do not know exactly how high this risk might be. At this time there is no treatment for these gene variants.

In the consent form we will ask you to indicate if you wish to be informed about variants in cancer genes. In the future we may ask you to confirm your decision. You can change your mind at any time. If you have a cancer gene variant and you have indicated that you wish to be informed, we will send you a letter inviting you to visit a Family Cancer Clinic to see a specialist doctor or genetic counsellor. In the meantime, we will also give you the contact details of a genetic counsellor that you may contact any time during business hours. At the Family Cancer Clinic, the genetics specialist can help you to think about what this might mean for you and your family, describe any processes for confirmatory genetic testing and discuss the screening and risk management options that may be available and support you as you learn about the gene variant. If you are worried about a family history of cancer, you can ask to be referred to the Family Cancer Clinic before the results of the research become available. Your GP can refer you to a local clinic.

- **You may have a gene variant that is not related to cancer**

There is a chance that whole genome sequencing may detect a gene variant that is not related to cancer. This is called an incidental finding. Incidental findings can sometimes be important to your future health and the health of your family (blood relatives) and future children. The findings can include gene variants that cause or may mean an increased risk of a serious health condition. These health problems may include but not be limited to heart conditions, dementia, high blood pressure and high cholesterol.

In the consent form we will ask you to indicate if you wish to be informed about incidental findings that may be important to your health and the health of your blood relatives. You may choose whether or not to be informed. In the future we may ask you to confirm your decision. You can change your mind at any time. If you choose to be informed we will send you a letter advising you that we have detected a genetic variant that may be important to your health. If you choose to learn more, we will arrange an appointment with an appropriate clinical specialist to explain more to you about what the genetic variant could mean for you and your family.

- **If you have a gene variant there may be insurance implications**

Statutory or contractual duties may require you to disclose results of genetic tests or analysis to third parties (for example, insurance companies, employers, financial and educational institutions), particularly where results provide information about health prospects. If the results

of your genetic tests are not available to you or you choose not to have the results given to you, then your future requests for insurance may not be affected by participating in this research.

Details of your family history are relevant in assessing your risk profile for certain forms of insurance. The ability to obtain private health insurance is not changed by your family history, genetic test results or health status. The Financial Services Council (FSC) has a voluntary agreement within its membership that any existing life, trauma or disability insurance that you may have will not be affected by your participation in the study.

Genetic information actually acquired by you as a result of your participation in this research may have implications for you (or your relatives') ability to obtain cover for certain risk rated insurance products offered either alone or as part of a superannuation product (eg insurance products providing cover for: life disability (income protection), trauma or any business or bank loans which require a policy for life (disability or trauma) and may impact upon the amount you pay for and scope of protection provided by such products.

- **There are minimal risks from having a blood sample taken**

In general there are minimal if any side effects from the blood taking procedure involved with participating in the study. Occasionally there may be some small pain or discomfort and a small bruise may form.

- **You may experience some psychological distress**

You may feel that some of the questions we ask are stressful or upsetting. If you do not wish to answer a question, you may skip it and go to the next question, or you may stop immediately. If you have a gene variant, support will be provided by a Family Cancer Clinic.

- **Genetic testing may reveal unexpected misattributed paternity or maternity**

In some testing situations, if a person's social parent is not their biological parent, genetic testing might reveal it. This can happen when certain types of tests are done on several members of the one family. If this is detected, the study will not disclose it to you unless required by law to do so, which is unlikely.

- **There is a small risk to your privacy due to stored data**

There is a small risk to your privacy because personal information is used in the record linkage process. We will supply your personal information (name, date of birth, address) to the relevant registry or databank so that they can identify you correctly. The registry will retrieve all the relevant associated information and send it back to the study. All safety measures have been put in place to ensure the confidentiality of your information. Strict guidelines regarding data transfer, storage and access will be adhered to.

We wish to link data with cancer registries and the Australian Institute of Health and Welfare to confirm cancer diagnoses and obtain other health related information.

9. What will happen to my test samples?

Your blood sample will be used for the purposes of this research project. Genetic material (DNA and RNA) will be extracted and sometimes long living cell lines will be established.

Your blood sample will be stored in a re-identifiable manner so that if we find any genetic results that may be significant to the health of you or your family we will be able to give you that information should you wish to learn about it.

Any tumour samples collected from pathology services will also be stored in a re-identifiable manner. All your samples will be stored indefinitely.

10. What is the potential impact on my family if I take part?

We will ask you to give us health information about your relatives. Any information you give us will be kept confidential. We will invite some of your first degree relatives to participate in the study. We will ask you to get their permission before contacting them. We will not contact your relatives without your permission. If the research discloses that one of your family members may be at risk of a life-threatening or serious illness for which treatment is available or pending, this information may, with the prior approval of a Human Research Ethics Committee, be offered by the study doctor to the family member, even if you as the participant do not consent to this.

11. Will I be given the results of this research project?
Your genetic test results important to health will be available to you should you wish. It is your decision whether you wish to be informed of these results. Before you decide if you wish to have your genetic test results, it is important that you read the information above regarding risks carefully so that you can make an informed decision and see a genetic counsellor if you so wish.

If we find a gene variant and you have indicated that you want to be informed about this information, we will send you a letter asking you to come to a familial cancer centre or another medical centre as needed. We will also give you the contact details of a genetic counsellor that you may contact any time during business hours. The genetic counsellor will be able to answer your questions and help you arrange your visit to the familial cancer centre.

Genetic information is complex and can be influenced by other factors including environment and lifestyle. Because genetic information is complex and sensitive, the results should be discussed with a clinical geneticist and genetic counsellor who can give you details that are relevant to you, answer your questions and discuss your concerns.

In the future, if during the course of this research project we discover new information that is important for your health care, you will be asked whether you wish to receive the results (this may require you to have the test repeated in a clinical laboratory). If you agree, we may contact you if such a situation arises.

14. Will I be informed of results of future research using my biospecimens?
As detailed in section 11, genetic results important to your health will be available should you wish.

15. Banking of health and other information
The health and other information we will collect and store in a bank will include your date of birth, contact details, marital status, education details, medical history, family tree and lifestyle information.

If you consent, information about you will also be obtained from your health records held at this and other health organisations for the purpose of this research. By signing the consent form you agree to the research team accessing these records for the purposes of this Cancer Risk study. We will collect information about you by reviewing your medical records and other hospital charts. The type of information we will be collecting from your medical record includes the results of tests you have (e.g. CT scans, blood tests) and the treatments you have received. With your permission, we may also access and link data from the following sources containing information about your health, medication and treatment:

- Hospital, pathology, emergency department, and Births, Deaths and Marriages Registry records;
- Medicare Benefits Scheme records (i.e. your visits to health professionals);

- Pharmaceutical Benefits Scheme and Repatriation Pharmaceutical Benefits Scheme records (i.e. your use of prescription medicines);
- Cancer registry records;
- Australian Institute of Health and Welfare records

As required by each data source, third parties may be involved in the record linkage process (for example the Centre for Health Record Linkage will be involved in linkage to the NSW Cancer Registry). All parties involved will be professional bodies adhering to the highest standards of confidentiality.

Before any data linkage occurs with external sources, separate approvals will be sought as appropriate. Approval will be sought from the NSW Population and Health Services REC before linkage to the NSW Cancer Registry occurs.

Any information used from these data sources will be treated completely confidentially and used only for the purposes of the study.

We will not use your personal health information for a different research project without the permission of a Human Research Ethics Committee. Once all personal identification is removed, the information might be used or released for other purposes without asking you. Results of the research project may be presented in public talks or written articles but information will not be presented that identifies the participant.

16. What will happen to information about me?

By signing the consent form you consent to the research team collecting and using personal information about you for the research project. All this information will be treated confidentially. Your personal information will be stored in an identifiable manner in the central electronic database of the study. The database is protected by the latest security measures and only authorised study staff will have password protected access. All hard copy records will be kept under locked conditions.

Your information will only be used for the purposes of this research project and it will only be disclosed with your permission, except as required by law.

Your health records and any information obtained during the research project may be subject to inspection (for the purpose of verifying the procedures and the data) by the relevant authorities and authorised representatives of the sponsor, or as required by law. By signing the Consent Form, you authorise release of, or access to, this confidential information to the relevant research personnel and regulatory authorities as noted above.

In accordance with relevant Australian and/or New South Wales privacy and other relevant laws, you have the right to request access to your information collected and stored by the study team. You also have the right to request that any information with which you disagree be corrected. Please contact the study team member named at the end of this document if you would like to access your information.

It is anticipated that the results of this research project will be published and/or presented in a variety of forums. In any publication and/or presentation, information will be provided in such a way that you cannot be identified, except with your express permission.

We will provide you with regular newsletters about the study if you indicate that you wish to receive them.

17. Complaints and compensation

If you suffer any injuries or complications as a result of this research project, you should contact the study team as soon as possible and you will be assisted with arranging appropriate medical

treatment. If you are eligible for Medicare, you can receive any medical treatment required to treat the injury or complication, free of charge, as a public patient in any Australian public hospital.

If you suffer any distress or psychological injury as a result of this research project, you should contact the study team as soon as possible. They will assist you in arranging appropriate treatment and support.

18. Who is organising and funding the research?

This study has been initiated by Professor David Thomas and is being conducted by the Garvan Institute of Medical Research. The study is funded by the NSW Office of Health and Medical Research.

By taking part in this research project you agree that samples of your blood or tissue (or data generated from analysis of these materials) may be provided to the Garvan Institute of Medical Research. The Garvan may directly or indirectly benefit financially from your samples or from knowledge acquired through analysis of your samples.

You will not benefit financially from your involvement in this research project even if, for example, your samples (or knowledge acquired from analysis of your samples) prove to be of commercial value to the Garvan.

In addition, if knowledge acquired through this research leads to discoveries that are of commercial value to the Garvan, the study doctors or their institutions, there will be no financial benefit to you or your family from these discoveries.

No member of the research team will receive a personal financial benefit from your involvement in this research project (other than their ordinary wages).

19. Who has reviewed the research project?

All research in Australia involving humans is reviewed by an independent group of people called a Human Research Ethics Committee (HREC). The ethical aspects of this research project have been approved by the HREC of St Vincent’s Hospital, Sydney (EC00140).

This project will be carried out according to the *National Statement on Ethical Conduct in Human Research (2007)*. This statement has been developed to protect the interests of people who agree to participate in human research studies.

20. Further information and who to contact

The person you may need to contact will depend on the nature of your query. If you want further information concerning this project you can contact the **Study Co-ordinator**, Dr Mandy Ballinger, on 9355 5806 or any of the following people.

Principal Investigator

Name	Professor David Thomas
Position	Director, the Kinghorn Cancer Centre, Garvan Institute of Med Res
Telephone	02 9355 5770
Email	d.thomas@garvan.org.au

For matters relating to research at the site at which you are participating, the details of the local site complaints are

Complaints person

Position	Research Office Manager
Telephone	02 8382 4960
Email	SVHS.Research@svha.org.au

If you have any complaints about any aspect of the project, the way it is being conducted or any questions about being a research participant in general, then you may contact

Reviewing HREC approving this research and HREC Executive Officer details

Reviewing HREC name	St Vincent's Hospital Sydney HREC

Local HREC Office contact (Single Site – Research Governance Officer)

Position	HREC Executive Officer
Telephone	02 8382 4960
Email	SVSH.research@SVHA.org.au

Consent Form

**Title**	**Genetic Cancer Risk in the Young Study**
**Short title**	**Cancer Risk Study**
**Protocol Number**	SVH file number 16/010
**Principal Investigator**	**Professor David Thomas**
**Site Investigator(s)**	Professor Allan Spigelman, Dr Mandy Ballinger
**Location**	St Vincent's Hospital Sydney

Declaration by Participant

I have read the Participant Information Sheet or someone has read it to me in a language that I understand.

I understand the purposes, procedures and risks of the research described in the project.

I have had an opportunity to ask questions and I am satisfied with the answers I have received.

I freely agree to participate in this research project as described and understand that I am free to withdraw at any time during the project without affecting my future care.

I understand that I will be given a signed copy of this document to keep.

I give permission for my doctors, other health professionals, hospitals or laboratories outside this hospital to release information to the Garvan Institute of Medical Research concerning my condition and treatment for the purposes of this project. I understand that such information will remain confidential.

I wish to receive the study newsletter
YES ☐ NO ☐

I wish to be informed if I am found to have a gene variant that causes cancer
YES ☐ NO ☐

I wish to be informed if I am found to have an incidental finding that may be important to my health
YES ☐ NO ☐

In the event of my death, any information important to health may be made known to relevant health professionals involved in my care and the care of other family members
YES ☐ NO ☐

In the event of my death, the information important to health may be made known to:

Name ___________________________________ Relationship _______________________

Contact details ____

In respect to the storage and use of my genetic samples, I give permission for the use of my DNA and/or tissue for the purpose of:

1. this research project YES ☐ NO ☐

I understand that I can withdraw my consent to participate in this research project by completing a "Withdrawal of Consent" form. I can also specify whether I wish to have my blood (and possible tumour) specimen, which has already collected and stored, deleted, destroyed or returned to me if it is still identifiable as mine.

Name of Participant (please	______________________________
Signature ______________________	Date ______________________

Name of Witness* to Participant's Signature (please print)	______________________________
Signature ______________________	Date ______________________

* Witness is not to be the investigator, a member of the study team or their delegate. In the event that an interpreter is used, the interpreter may not act as a witness to the consent process. Witness must be 18 years or older.

Declaration by person performing the informed consent discussion

I have given a verbal explanation of the research project, its procedures and risks and I believe that the participant has understood that explanation.

Name (please print)	______________________________
Signature ______________________	Date ______________________

Note: All parties signing the consent section must date their own signature.

ST VINCENT'S HOSPITAL
SYDNEY
A FACILITY OF ST VINCENT'S HEALTH AUSTRALIA

Form for Withdrawal of Participation

**Title**	Genetic Cancer Risk in the Young Study
**Short Title**	Cancer Risk Study
**Protocol Number**	SVH file number 16/010
**Coordinating Principal Investigator/ Principal Investigator**	Professor David Thomas
**Site Investigator(s)**	Professor Allan Spigelman, Dr Mandy Ballinger
**Location**	St Vincent's Hospital Sydney

Declaration by Participant

I wish to withdraw from participation in the above research project and understand that such withdrawal will not affect my routine treatment, my relationship with those treating me or my relationship with St Vincent's Hospital Sydney.

I request that all my blood and/or tumour samples collected and banked be deleted, destroyed or returned to me if it is still identifiable.
YES ☐ NO ☐

Name of Participant (please print) ______________________________

Signature ______________________ Date ______________________

In the event that the participant's decision to withdraw is communicated verbally, the Study Doctor/Senior Researcher will need to provide a description of the circumstances below.

Declaration by Study Doctor/Senior Researcher†

I have given a verbal explanation of the implications of withdrawal from the research project and I believe that the participant has understood that explanation.

Name of Study Doctor/
Senior Researcher† (please print) ______________________________

Signature ______________________ Date ______________________

† A senior member of the research team must provide the explanation of, and information concerning, withdrawal from the research project.

Note: All parties signing the consent section must date their own signature.
