# Supplementary material for: Cancer Patient Experience of Uncertainty While Waiting for Genome Sequencing Results
Source: Front Psychol. 2021 Apr 22;12:647502. doi: 10.3389/fpsyg.2021.647502 (PMC8100530; doi:10.3389/fpsyg.2021.647502)
Supplement: Supplementary file 2 [file Data_Sheet_2.PDF]

RISK Study: Baseline (T0) interview guide

To explore participant expectations and understanding of whole genome sequencing.

Introduction:

Introduce self – working on the RISK study

Are you still ok to do this interview now?

Purpose - to discuss your experience of the study - no right or wrong ways of experiencing this, we are interested in your perspective

Will take no longer than 30 minutes - can stop the interview anytime you like. If you do not understand any of the questions, I will try my best to explain it.

Recording the interviews to help us remember and analyse everyone's responses; your recording will be anonymised and your identity will not be reported. Are you happy for me to record this interview?

Do you have any questions before we begin?

Questions: Participants and family members

1. **Tell me a bit about yourself. How did you come to know about the study?**

   Note to interviewer:
   - Want to know who told them about the study – recruited in person at TKCC; via genetic counsellor

2. **Can you tell me what you know about the blood test you had for the study to look at your genes (whole genome sequencing)? (if anything)**

   Probe understanding of:
   - How testing is done?
   - What is its potential to find anything useful?
   - Likely costs for tests/subsequent interventions if offered?
   - Likelihood of finding incidental findings?

3. **Had you heard about whole genome sequencing before the study? Have you had any previous experience of genetic testing before this study?**

   Probe:
   - What; why?
   - How did you feel that test and result?
   - Comparison of their experiences for the two types of testing

4. **Why do you think you were offered this test?**

5. **Whole genome sequencing is a blood test which involves mapping all your genes in one test, to look for gene variants? What do you think about it?**

Note for interviewer: Looking for knowledge and understanding of the implications, i.e., inheritance, risk assessment.
Probe for understanding:
- Do you understand what this means?

6. **What do you see as the benefits (if any) of whole genome sequencing?**

Probe:
- What do you hope to gain from this test (if anything)?
- What do you think the test will tell you?
- What types of information do you expect to learn (was there a family history)?

7. **What worries you about it?**

Note for interviewer: Looking for examples of financial, emotional and practical drawbacks, like third party access to information, impact on insurance.

Probe:
- How important are these worries to you?

8. **Why did you decide to participate?**

Note for interviewer: Interested in rationale for participating, particularly in relation to any drawbacks the participant listed in Question 7.
Probe:
- To help medical research; learn about your genetics; help medical care; because I have been in another similar study
- How did you weigh up your decision to participate (how did the benefits and worries impact the decision)?

9. **Do you think whole genome sequencing should be offered to people with a family history of cancer like you?**

Probe:
- Why; why not?
- Is there anyone else that should be offered the test?
- Views on innovative tests and the need for evidence to validate before making clinical practice?
- How the information is used

10. If a public healthcare system can't fund whole genome sequencing for everyone, how do you think we should decide who gets these tests?

Probe for perspective on:

- Intuition about need; how they define need
- Technology

11. Before deciding to have this test, do you think people should be provided with some resources and information? If so, what and how? How long do you think they should have to decide about the test before it takes place?

Probe for perspective on:

- What are some of the things that you would like to see as part of the process for decision making about this kind of test?
- Information formats - written information; online video; pictures (more controversially, whether this is something people should give permission for, or whether it should be given to everyone)
- What level of detail is needed to make a decision that they feel comfortable with?
- What else do you need besides information? Are the details important, or trust of Doctor? (What is ethically relevant to the participant?)
- Would you value working more in partnership with your healthcare professional
- What support do people need to make a decision?
- Time to reflect – minimum timeframe?

12. I see from your consent form that you decided to receive X (i.e. all genetic information with medical relevance, cancer actionable information only, etc) results. Can you tell me why you made that decision? Do you think you should receive all the possible information that your test might find, or just information relevant to your cancer?

Probe for perspective on:

- What about the timeframe? Should the information be staggered over time (e.g., providing information now that is more relevant to your cancer, and other information if it is available at a later point in time)
- Do you think it is possible to get too much information/what is the right amount of information?
- Are there any downsides to information?

13. Do you think it is okay to participate in this kind of research but choose to not receive any results at all?

Probe:

- What about contribution to knowledge?

14. **Should people be given genetic information, say if it could lead to life saving treatment, even if they said they did not want to receive it?**

15. **This won't happen to you in this study, but how do you think you would react if you were told that changes in your genes had been found, but no-one currently knew what they meant?**

Probe:
- Resilience under uncertainty
- What could be done to mitigate anxiety?

16. **How do you generally deal with uncertainty in your life?**

Example: going on a car trip in an old car where something is likely to go wrong, but you don't know when.

Probe:
- What sort of uncertainty are you experiencing about whole genome sequencing?
- What is the hardest to cope with?
- What would help you manage the uncertainty?

17. **Who did you talk to about this study?**

Probe:
- Who? – family; friends; GP; Oncologist
- Why/why not?
- When?
- What did you say? What did you tell them?
- What sort of support would you like in having these types of conversations?

18. **Have you thought about if you would talk to other family members if a genetic change is found that can be inherited in the family? How would you go about doing that? Do you expect any challenges?**

Probe:
- Why/why not?
- Which relatives?
- When? How?
- What would you say?
- How are you feeling about that conversation?
- What sort of support would you like with these conversations?

19. **Is there anything else you would like to say about whole genome sequencing? Do you have any advice about how this topic should be discussed with other people?**
