# Supplementary material for: Cancer Patient Experience of Uncertainty While Waiting for Genome Sequencing Results
Source: Front Psychol. 2021 Apr 22;12:647502. doi: 10.3389/fpsyg.2021.647502 (PMC8100530; doi:10.3389/fpsyg.2021.647502)
Supplement: Supplementary file 3 [file Data_Sheet_3.PDF]

Genetic Cancer Risk in the Young Study

Short title: Cancer Risk Study

Participant Questionnaire 1

As part of the Cancer Risk Study, we hope to better understand the impact of new genetic testing technologies on emotions and behaviour, health costs and outcomes. Thus we are inviting you to complete this questionnaire as someone who is participating in the Cancer Risk Study. In this questionnaire, we are asking about all possible responses to whole genome sequencing. Many of these may not be relevant to you, but it is important for us to get a clear picture of everyone's experiences.

We would greatly appreciate if you completed this questionnaire within the next 1-2 weeks. It will take about 20 minutes to complete. Once completed the questionnaire can be submitted online or returned using the reply paid envelope provided.

Your responses will be kept confidential and your identity will not be revealed in any reports or presentations. Information you provide will not be shared with any health professionals involved in your care.

Participation in this study is voluntary and you can withdraw from the study at any time. If you would prefer not to participate in the study, please let us know by contacting the study coordinator (Mandy Ballinger, 02 9355 5806). Your participation (or non-participation) will not affect your relationship with any treating doctors, other health professionals or researchers involved with the Cancer Risk Study.

If you have any questions about filling in the questionnaire, or the study in general, please call the project officer Christine Napier on (02 9355 5839).

Participant number:

			--				

Date issued:

Date completed:

1. **What is your date of birth?**

day / month / year
☐☐ / ☐☐ / ☐☐☐☐

2. **What is today's date?**

day / month / year
☐☐ / ☐☐ / ☐☐☐☐

3. **What was your sex at birth?**

☐ Male ☐ Female

4. **What is your current marital status?**

☐ Married
☐ Single
☐ De facto/living with a partner
☐ Separated
☐ Divorced
☐ Widowed

5. **What is the postcode for the suburb you live in?**

☐☐☐☐

6. **What was the highest level of education that you completed?**

☐ Primary school (some or all)
☐ Secondary school – year 7 or 8
☐ Secondary school – year 9 or 10
☐ Secondary school – year 11 or 12
☐ Vocational training
☐ University – did not graduate
☐ University – graduated
☐ Don't know

7. **In which country were you, your parents and your grandparents born?**
Please write the country

	Country
You	
Your mother	
Your mother's mother	
Your mother's father	
Your father	
Your father's mother	
Your father's father	

8. **If you were not born in Australia, for how many years have you lived in Australia?**

☐☐ years

9. What is your ancestry?

☐ Australian ☐ Lebanese ☐ Italian ☐ Vietnamese
☐ English ☐ Scottish ☐ German ☐ Filipino
☐ Chinese ☐ Maltese ☐ Greek ☐ Indian
☐ Irish ☐ Polish ☐ Dutch ☐ Croatian
☐ Other *(please specify)* ______________________

10. Do you speak a language other than English at home?

☐ Yes. Please specify ______________________
☐ No

11. How many biological children do you have?

☐☐ children

12. How old were you when you had your first child?

☐☐ years old

13. How tall are you without shoes on?

☐ cm **OR** ☐ feet ☐ inches

14. What is your current weight?

☐ kg **OR** ☐ stone ☐ lbs

15. Has a doctor ever told you that you have cancer, leukaemia or a malignant tumour?

☐ Yes
☐ No – go to question 16
☐ Don't know – go to question 16

What was the type of cancer and when was this first diagnosed? Just write 'I don't know' if you can't fill in the details.

	Type of cancer	*Age at diagnosis*
First cancer		
Second cancer		
Third cancer		
Fourth cancer		

16. Have other members of your family been diagnosed with any types of cancer?

☐ Yes
☐ No – go to question 17
☐ Don't know – go to question 17

Who has had cancer, what was the type of cancer and when was this first diagnosed? Just write 'I don't know' if you can't fill in the details.

Relationship to you *Please indicate if the relation is on your mother's or father's side of the family*	*Type of cancer*	*Age at diagnosis*

17. A family cancer clinic focuses on hereditary risk factors for cancer. Have you ever been to a family cancer clinic?

☐ Yes
☐ No – go to question 18
☐ Don't know – go to question 18

Which family cancer clinic did you go to?

Clinic, City

18. Have you ever had genetic testing?

☐ Yes
☐ No – go to question 19
☐ Don't know – go to question 19

What condition were you tested for?

Condition

19. Have you ever had radiation therapy?

☐ Yes
☐ No – go to question 20
☐ Don't know – go to question 20

Can you please provide some information about the radiation therapy?

Reason for treatment	*Treatment centre*	*Treatment date*

20. Have you ever had chemotherapy?

☐ Yes
☐ No – go to question 21
☐ Don't know – go to question 21

Can you please provide some information about the chemotherapy?

Reason for treatment	*Treatment centre*	*Treatment date*

21. Have you ever been a regular smoker?

☐ Yes
☐ No – go to question 22

How old were you when you started smoking regularly?

☐☐ years old

Are you a regular smoker now?

☐ Yes
☐ No

If No – how old were you when you stopped smoking regularly?

☐☐ years old

About how much do you / did you smoke on average each day?

☐☐ cigarettes per day
☐☐ pipes and cigars per day

22. About how many alcoholic drinks do you have each week?
One drink = a glass of wine, middy of beer or nip of spirits (put "0" if you do not drink, or have less than one drink each week)

☐ number of alcoholic drinks each week

23. On how many days each week do you usually drink alcohol?

☐ days each week

24. What has been your main occupation?

Main occupation

☐

Genes contain the instructions for our body to function properly. Changes in genes, called variants, may contribute to causing cancer. The blood test you have had for whole genome sequencing was taken to look for these cancer **gene variants**. Whole genome sequencing test results may indicate that you and / or your blood family members are at higher risk of developing cancer than other people in the community. Knowing this can guide interventions to reduce your risk of cancer.

25. How important is it to you to learn about gene variants that may increase your chances of getting cancer?

❑ Not at all important
❑ A little bit important
❑ Somewhat important
❑ Moderately important
❑ Very important

26. How important is it to you to learn more about how your lifestyle, such as exercise, smoking and diet, affects your chance of getting certain diseases?

❑ Not at all important
❑ A little bit important
❑ Somewhat important
❑ Moderately important
❑ Very important

27. How much do you think inherited genes determine whether or not a person will develop cancer?

❑ Not at all
❑ A little bit
❑ Somewhat
❑ A lot
❑ Completely

28. How much do you think lifestyle, such as exercise, smoking and diet, determine whether or not a person will develop cancer?

❑ Not at all
❑ A little bit
❑ Somewhat
❑ A lot
❑ Completely

29. How much personal control do you feel you have over whether you develop cancer in the future?

❑ No control
❑ Little control
❑ Some control
❑ Moderate control
❑ A lot of control

Whole genome sequencing is a blood test which involves mapping the sequence of all of your genes in one test, in order to look for gene variants.

The next questions ask what you know about whole genome sequencing.

Please tick ONE ANSWER FOR EACH QUESTION (i.e. the answer you think is the most accurate).

30. If one of your parents has a gene variant, the chance of inheriting that gene variant is:

❑ 25% (1 in 4 chances)
❑ 50% (1 in 2 chances)
❑ 100%
❑ I don't know
❑ Other *(please explain)*

31. Whole genome sequencing involves testing:

❑ 200 genes
❑ 2,000 genes
❑ 20,000 genes
❑ I don't know
❑ Other *(please explain)*

32. The results of whole genome sequencing are helpful for making decisions about future cancer risks:

❑ Never
❑ Sometimes
❑ Frequently
❑ Always
❑ I don't know

33. The likelihood of finding a gene variant to guide prevention depends on the type of cancer.

❑ True
❑ False
❑ I don't know

34. Whole genome sequencing is helpful for understanding the risk of:

❑ No types of cancer
❑ Some types of cancer
❑ Most types of cancer
❑ All types of cancer
❑ I don't know

35. Sometimes we find a gene variant we know nothing about:

❑ True
❑ False
❑ I don't know

36. Sometimes cancer screening or preventative surgery, or cancer treatment, can be offered to people with a gene variant. The costs of this would be :

❑ Covered in full by Medicare (at no cost to the patient)
❑ Only available through a clinical trial (at no cost to the patient)
❑ Only available privately (at the patient's cost)
❑ I don't know

37. From where have you learned most about whole genome sequencing?

❑ My oncologist
❑ The researchers doing this study
❑ School or university
❑ TV
❑ Online
❑ Other *(please specify)* ___________________________________

If whole genome sequencing was available to predict cancer do you think it should be done on:

38. *Your relatives?*

❑ Yes
❑ No

If yes, at what age? (please choose one answer only)

❑ Newborn babies and children (up to about 16 years), ***without*** parental consent
❑ Newborn babies and children, requiring parental consent
❑ Teenagers (16-19 years), ***without*** their consent
❑ Teenagers (16-19 years), only with their consent
❑ Adults between ages 20-30 years, ***without*** their consent
❑ Adults between ages 20-30 years, only with their consent
❑ Adults 30 years and over, ***without*** their consent
❑ Adults 30 years and over, only with their consent

39. *All people?*

❑ Yes
❑ No

If yes, at what age? (please choose one answer only)

❑ Newborn babies and children (up to about 16 years), ***without*** parental consent
❑ Newborn babies and children, requiring parental consent
❑ Teenagers (16-19 years), ***without*** their consent
❑ Teenagers (16-19 years), only with their consent
❑ Adults between ages 20-30 years, ***without*** their consent
❑ Adults between ages 20-30 years, only with their consent
❑ Adults 30 years and over, ***without*** their consent
❑ Adults 30 years and over, only with their consent

40. ***Anyone who requests it?***

❑ Yes
❑ No

If yes, at what age? (please choose one answer only)

❑ Newborn babies and children (up to about 16 years), ***without*** parental consent
❑ Newborn babies and children, requiring parental consent
❑ Teenagers (16-19 years), ***without*** their consent
❑ Teenagers (16-19 years), only with their consent
❑ Adults between ages 20-30 years, ***without*** their consent
❑ Adults between ages 20-30 years, only with their consent
❑ Adults 30 years and over, ***without*** their consent
❑ Adults 30 years and over, only with their consent

The next question asks how *confident* you are about having whole genome sequencing. Please tick one of the boxes to show how much you agree with the statement.

41. I am confident that if I did have whole genome sequencing, I would be able to:

	**Strongly disagree**	**Disagree**	**Neither agree or disagree**	**Agree**	**Strongly agree**
a. Cope if a gene variant indicating that I and my family are at risk of some cancer, was found	❑	❑	❑	❑	❑
b. Cope if a gene variant indicating that I and my family are at risk of some cancers, was NOT found	❑	❑	❑	❑	❑
c. Cope if a gene variant was found that no-one knew anything about	❑	❑	❑	❑	❑
d. Cope with telling other members of my family about an inherited gene variant	❑	❑	❑	❑	❑

The following question asks you about whether you think you would make any changes in your lifestyle *if you were told you had inherited gene variants which increase your risk of cancer*. Please tick one of the boxes to show how much you agree or disagree with each statement.

42. If I knew I had inherited genes which increase my risk of cancer:

	Strongly disagree	Disagree	Neither agree or disagree	Agree	Strongly agree
a. I would be more careful with my diet	❑	❑	❑	❑	❑
b. I would exercise more	❑	❑	❑	❑	❑
c. I would change the amount of alcohol I drink	❑	❑	❑	❑	❑
d. I would reduce the amount I smoke	❑	❑	❑	❑	❑
e. I would seek more information	❑	❑	❑	❑	❑
f. I would learn to manage stress better	❑	❑	❑	❑	❑
g. I would want more health screening	❑	❑	❑	❑	❑

The next questions ask about whether you would have whole genome sequencing, and how much you would be prepared to pay, *depending on how likely finding information to guide prevention is.*

As part of your participation in this study, you will be provided with whole genome sequencing at no cost. However, we are interested to know whether people would consider having whole genome sequencing if they had to pay for it, as part of routine care. For questions 43-48, please imagine you are being offered whole genome sequencing by your doctor.

43. If whole genome sequencing found a gene variant to guide prevention of cancer ***in about 1 in 100 people (1%),*** **would you have the test?**

❑ Yes
❑ No

What is the most money you would be prepared to pay for it, if this was your 'out of pocket' expense? (Remember, your test is at no cost due to your participation in this research).

Highest amount you would be prepared to pay

❑ $0
❑ $300
❑ $1,000
❑ $3,000
❑ $10,000

44. If whole genome sequencing found a gene variant to guide prevention of cancer ***in about 10 in 100 people (10%),*** **would you have the test?**

❑ Yes
❑ No

What is the most money you would be prepared to pay for it, if this was your 'out of pocket' expense?

Highest amount you would be prepared to pay

❑ $0
❑ $300
❑ $1,000
❑ $3,000
❑ $10,000

45. If whole genome sequencing found a gene variant to guide prevention of cancer ***in about 20 in 100 people (20%)***, **would you have the test?**

❑ Yes
❑ No

What is the most money you would be prepared to pay for it, if this was your 'out of pocket' expense?

Highest amount you would be prepared to pay

❑ $0
❑ $300
❑ $1,000
❑ $3,000
❑ $10,000

46. If whole genome sequencing found a gene variant to guide prevention of cancer ***in about 30 in 100 people (30%)***, **would you have the test?**

❑ Yes
❑ No

What is the most money you would be prepared to pay for it, if this was your 'out of pocket' expense?

Highest amount you would be prepared to pay

❑ $0
❑ $300
❑ $1,000
❑ $3,000
❑ $10,000

47. If whole genome sequencing found a gene variant to guide prevention of cancer ***in about 40 in 100 people (40%)*****, would you have the test?**

❑ Yes
❑ No

What is the most money you would be prepared to pay for it, if this was your 'out of pocket' expense?

Highest amount you would be prepared to pay

❑ $0
❑ $300
❑ $1,000
❑ $3,000
❑ $10,000

48. If whole genome sequencing found a gene variant to guide prevention of cancer ***in about 50 in 100 people (50%)*****, would you have the test?**

❑ Yes
❑ No

What is the most money you would be prepared to pay for it, if this was your 'out of pocket' expense?

Highest amount you would be prepared to pay

❑ $0
❑ $300
❑ $1,000
❑ $3,000
❑ $10,000

49. This study is specifically trying to identify gene variants associated with cancer. As a result of participating in genetic research it is possible that gene variants with associations to other conditions may be found.

What sort of genetic conditions do you think people would like to be informed about when they take part in genetic research?

	**Yes**	**No**	**Maybe**	**Don't know**
a. Known genetic conditions caused by one gene, for which there is *no prevention* (e.g. diet, exercise) or *treatment* that can *change the risk* (e.g. inherited blindness)	❑	❑	❑	❑

	Yes	No	Maybe	Don't know
b. Known genetic conditions caused by one gene, for which there is *prevention* (e.g. screening) or *treatment* that can *change the risk* (e.g. breast or bowel cancer)	❑	❑	❑	❑
c. Known genetic conditions caused by many genes, which can have a major impact on health, for which there is *treatment as well as lifestyle factors* (e.g. diet, exercise, stopping smoking) which can *modify the risk* (e.g. cancer, heart disease)	❑	❑	❑	❑
d. Known genetic conditions caused by many genes, which usually have a lower impact on health, for which there is *treatment as well as lifestyle factors* which can *modify the risk* (e.g. asthma)	❑	❑	❑	❑

50. People who are at high risk of cancer often worry about the possibility of developing cancer.

Most people who have had cancer worry about the possibility of a recurrence of the cancer. By recurrence we mean the possibility that the cancer will return or progress in the same place or in another part of your body.

For each question please tick the box for the answer that best reflects how you felt in THE PAST MONTH.

a. How often have you worried about the possibility of developing cancer / having a recurrence of cancer?

0	1	2	3	4	5	6	7	8	9	10

None of the time — All of the time

b. To what extent does worry about developing cancer / having a recurrence of cancer spill over or intrude on your thoughts and activities?

0	1	2	3	4	5	6	7	8	9	10

Not at all — A great deal

c. How emotionally upset or distressed have you been about the possibility of developing cancer / having a recurrence of cancer?

0	1	2	3	4	5	6	7	8	9	10

Not at all — A great deal

For the next questions, compare yourself with someone who has the same risk of cancer, or the same cancer as you.

d. Compared to an average person of the same age and gender as you, what do you think are your chances of developing cancer or having a recurrence of cancer?

Much lower	Lower	Same	Higher	Much higher
❑	❑	❑	❑	❑

e. If you were to express this as a number, or percentage, what would you say? Please place a vertical mark on the line below, where 0% = I am certain that I will not develop / have a recurrence of cancer; and 100% = I am certain that I will develop / have a recurrence of cancer.

0%	50%	100%
**No chance of cancer progression or developing cancer**	**50-50 chance of progression or developing cancer**	**Will definitely progress or I will develop another cancer**

f. Compared to an average person of the same age and gender as you, what do you think are your chances of having a gene variant that puts you at increased cancer risk?

Much lower	Lower	Same	Higher	Much higher
❑	❑	❑	❑	❑

The next question asks about how you cope with uncertainty.

51. Please indicate how much you agree or disagree with each statement by ticking the box that best represents your views.

	Strongly disagree	Disagree	Neither agree or disagree	Agree	Strongly agree
a. I would rather receive my whole genome sequencing test results, and be certain about my future health, even if the result is bad news	❑	❑	❑	❑	❑
b. I would like to know now if I am likely to develop (another) cancer so I can get used to the news	❑	❑	❑	❑	❑
c. If I didn’t receive my whole genome sequencing test results I would always be wondering whether I was going to develop (another) cancer	❑	❑	❑	❑	❑

	Strongly disagree	Disagree	Neither agree or disagree	Agree	Strongly agree
d. The relief I would get from getting a low risk result makes it worth the risk that the result is bad	❑	❑	❑	❑	❑
e. I think it is tempting fate to ask questions about future illness	❑	❑	❑	❑	❑
f. I would rather live with uncertainty, than find out I was going to develop (another) cancer	❑	❑	❑	❑	❑
g. Knowing the result of my whole genome sequencing test would mean I felt more in control	❑	❑	❑	❑	❑
h. It is better to know that I will develop (another) cancer, even if I can't prevent it	❑	❑	❑	❑	❑

52. Considering your decision to have whole genome sequencing, please indicate to what extent each statement is true for you AT THIS TIME.

	Strongly disagree	Disagree	Neither agree or disagree	Agree	Strongly agree
a. I am satisfied that I am adequately informed about the issues important to my decision	❑	❑	❑	❑	❑
b. The decision I made was the best decision possible for me personally	❑	❑	❑	❑	❑
c. I am satisfied that my decision was consistent with my personal values	❑	❑	❑	❑	❑
d. I expect to successfully carry out the decision I made	❑	❑	❑	❑	❑
e. I am satisfied that this was my decision to make	❑	❑	❑	❑	❑
f. I am satisfied with my decision	❑	❑	❑	❑	❑

53. Under each heading, please tick the ONE box that best describes your health TODAY.

a. MOBILITY

❑	I have no problems with walking around
❑	I have slight problems with walking around
❑	I have moderate problems with walking around
❑	I have severe problems with walking around
❑	I am unable to walk around

b. PERSONAL CARE

❑	I have no problems with washing or dressing myself
❑	I have slight problems with washing or dressing myself
❑	I have moderate problems with washing or dressing myself
❑	I have severe problems with washing or dressing myself
❑	I am unable to wash or dress myself

c. USUAL ACTIVITIES (e.g. work, study, housework, family or leisure activities)

❑	I have no problems doing my usual activities
❑	I have slight problems doing my usual activities
❑	I have moderate problems doing my usual activities
❑	I have severe problems doing my usual activities
❑	I am unable to do my usual activities

d. PAIN / DISCOMFORT

❑	I have no pain or discomfort
❑	I have slight pain or discomfort
❑	I have moderate pain or discomfort
❑	I have severe pain or discomfort
❑	I have extreme pain or discomfort

e. ANXIETY / DEPRESSION

❑	I am not anxious or depressed
❑	I am slightly anxious or depressed
❑	I am moderately anxious or depressed
❑	I am severely anxious or depressed
❑	I am extremely anxious or depressed

54. We would like to know how good or bad your health is TODAY.

- This scale is numbered from 0 to 100
- 100 means the <u>best</u> health you can imagine, 0 means the <u>worst</u> health you can imagine
- Mark an X on the scale to indicate how your health is **TODAY**
- Now, please write the number you marked on the scale in the box below

YOUR HEALTH TODAY = ☐

The best health you can imagine

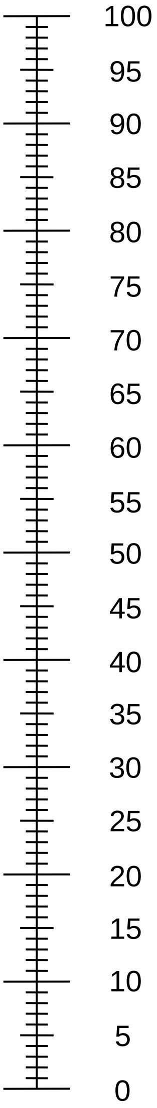

The worst health you can imagine

55. How much energy do you have to do the things you want to do? I am

❑	Always full of energy
❑	Usually full of energy
❑	Occasionally energetic
❑	Usually tired and lacking energy
❑	Always tired and lacking energy

56. How often do you feel socially excluded or left out?

❑	Never
❑	Rarely
❑	Sometimes
❑	Often
❑	Always

57. How easy or difficult is it for you to get around by yourself outside your place of residence (e.g. to go shopping, visiting)?

- ❑ Getting around is enjoyable and easy
- ❑ I have no difficulty getting around outside my place of residence
- ❑ A little difficulty
- ❑ Moderate difficulty
- ❑ A lot of difficulty
- ❑ I cannot get around unless somebody is there to help me

58. Does your health affect your role in your community (e.g. residential, sporting, church or cultural activities)?

- ❑ My role in the community is unaffected by my health
- ❑ There are some parts of my community role I cannot carry out
- ❑ There are many parts of my community role I cannot carry out
- ❑ I cannot carry out any part of my community role

59. How often do you feel sad?

- ❑ Never
- ❑ Rarely
- ❑ Some of the time
- ❑ Usually
- ❑ Nearly all the time

60. How often do you experience serious pain? I experience it

- ❑ Very rarely
- ❑ Less than once a week
- ❑ Three to four times a week
- ❑ Most of the time

61. How much confidence do you have in yourself?

- ❑ Complete confidence
- ❑ A lot
- ❑ A moderate amount
- ❑ A little
- ❑ None at all

62. Do you normally feel calm and tranquil or agitated? I am

- ❑ Always calm and tranquil
- ❑ Usually calm and tranquil
- ❑ Sometimes calm and tranquil, sometimes agitated
- ❑ Usually agitated
- ❑ Always agitated

63. Does your health affect your relationship with your family?

- ❑ My role in the family is unaffected by my health
- ❑ There are some parts of my family role I cannot carry out
- ❑ There are many parts of my family role I cannot carry out
- ❑ I cannot carry out any part of my family role

64. How satisfying are your close relationships (family and friends)?

- ❑ Very satisfying
- ❑ Satisfying
- ❑ Neither satisfying nor dissatisfying
- ❑ Dissatisfying
- ❑ Unpleasant
- ❑ Very unpleasant

65. How well do you communicate with others (talking, signing, texting, being understood by others and understanding them)?

❑	I have no trouble being understood
❑	I have some difficulty being understood by people who do not know me
❑	I am understood only by people who know me
❑	I cannot adequately communicate with others

66. How often do you have trouble sleeping?

❑	Never
❑	Almost never
❑	Sometimes
❑	Often
❑	All the time

67. How often do you feel worthless?

❑	Never
❑	Almost never
❑	Sometimes
❑	Usually
❑	Always

68. How often do you feel angry?

❑	Never
❑	Almost never
❑	Sometimes
❑	Often
❑	All the time

69. How easy or difficult is it for you to move around (using any aids or equipment you need e.g. a wheelchair, frame or stick)?

❑	I am very mobile
❑	I have no difficulty with mobility
❑	I have some difficulty with mobility (for example, going uphill)
❑	I have difficulty with mobility. I can go short distances only.
❑	I have a lot of difficulty with mobility. I need someone to help me.
❑	I am bedridden

70. Do you ever feel like hurting yourself?

❑	Never
❑	Rarely
❑	Sometimes
❑	Often
❑	All the time

71. How enthusiastic do you feel?

❑	Extremely
❑	Very
❑	Somewhat
❑	Not much
❑	Not at all

72. How often did you feel worried in the last seven days?

❑	Never
❑	Occasionally
❑	Sometimes
❑	Often
❑	All the time

73. How difficult is it for you to wash, toilet, dress yourself, eat or care for your appearance?

- ❑ These things are very easy for me to do
- ❑ I have no real difficulty in doing these things
- ❑ I find some of these things difficult, but I manage to do them on my own
- ❑ Many of these things are difficult, and I need help to do them
- ❑ I cannot do these things by myself at all

74. How often do you feel happy?

- ❑ All the time
- ❑ Mostly
- ❑ Sometimes
- ❑ Almost never
- ❑ Never

75. How much do you feel you can cope with life's problems?

- ❑ Completely
- ❑ Mostly
- ❑ Partly
- ❑ Very little
- ❑ Not at all

76. How much pain or discomfort do you experience:

- ❑ None at all
- ❑ I have moderate pain
- ❑ I suffer from severe pain
- ❑ I suffer unbearable pain

77. How much do you enjoy your close relationships (family and friends)?

- ❑ Immensely
- ❑ A lot
- ❑ A little
- ❑ Not much
- ❑ I hate it

78. How often does pain interfere with your usual activities?

- ❑ Never
- ❑ Rarely
- ❑ Sometimes
- ❑ Often
- ❑ Always

79. How often do you feel pleasure?

- ❑ Always
- ❑ Usually
- ❑ Sometimes
- ❑ Almost never
- ❑ Never

80. How much of a burden do you feel you are to other people?

- ❑ Not at all
- ❑ A little
- ❑ A moderate amount
- ❑ A lot
- ❑ Totally

81. How content are you with your life?

- ❑ Extremely
- ❑ Mainly
- ❑ Moderately
- ❑ Slightly
- ❑ Not at all

82. How well can you see (using your glasses or contact lenses if they are needed)?

- ❑ I have excellent sight
- ❑ I see normally
- ❑ I have some difficulty seeing things sharply (e.g. small print, objects in the distance, or watching television)
- ❑ I have a lot of difficulty seeing sharply
- ❑ I only see general shapes
- ❑ I am completely blind

83. How often do you feel in control of your life?

- ❑ Always
- ❑ Mostly
- ❑ Sometimes
- ❑ Only occasionally
- ❑ Never

84. How much help do you need with jobs around your place of residence (e.g. preparing food, cleaning, gardening)?

- ❑ I can do all these tasks very easily without any help
- ❑ I can do these tasks relatively easily without help
- ❑ I can do these tasks only very slowly without help
- ❑ I cannot do most of these tasks unless I have help
- ❑ I can do none of these tasks by myself

85. How often do you feel socially isolated?

- ❑ Never
- ❑ Rarely
- ❑ Sometimes
- ❑ Often
- ❑ Always

86. How well can you hear (using your hearing aid if needed)?

- ❑ I have excellent hearing
- ❑ I hear normally
- ❑ I have some difficulty hearing or I do not hear clearly (e.g. when there is background noise)
- ❑ I have difficulty hearing things clearly. Often I do not understand what is said. I usually do not take part in conversations because I cannot hear what is said.
- ❑ I hear very little
- ❑ I am completely deaf

87. How often do you feel depressed?

- ❑ Never
- ❑ Almost never
- ❑ Sometimes
- ❑ Often
- ❑ Very often
- ❑ All the time

88. How happy are you with your close and intimate relationships?

- ❑ Very happy
- ❑ Generally happy
- ❑ Neither happy nor unhappy
- ❑ Generally unhappy
- ❑ Very unhappy

89. How often did you feel in despair in the last seven days?

- ❑ Never
- ❑ Occasionally
- ❑ Sometimes
- ❑ Often
- ❑ All the time

THANK YOU FOR COMPLETING THIS QUESTIONNAIRE
