# Supplementary material for: Cancer Patient Experience of Uncertainty While Waiting for Genome Sequencing Results
Source: Front Psychol. 2021 Apr 22;12:647502. doi: 10.3389/fpsyg.2021.647502 (PMC8100530; doi:10.3389/fpsyg.2021.647502)
Supplement: Supplementary file 4 [file Data_Sheet_4.PDF]

RisC Study: 3-month follow up (T1) interview guide

Introduction:

Introduce self – working on the RisC study

Are you still ok to do this interview now?

Purpose - to discuss your experience of the study - no right or wrong ways of experiencing this, we are interested in your perspective

Will take no longer than 30 minutes - can stop the interview anytime you like. If you do not understand any of the questions, I will try my best to explain it.

Recording the interviews to help us remember and analyse everyone's responses; your recording will be anonymised and your identity will not be reported. Are you happy for me to record this interview?

Do you have any questions before we begin?

Questions: Probands

1. **Tell me a bit about yourself and how you came to know about the study?**

2. **Can you tell me what you remember about the study? Has anything changed for you since you started the study?**

Probe understanding of:

- Can you tell me what you know, if anything, about the blood test that you had for the study to look at your genes?
- Do you think you understood what whole genome sequencing was when you started the study?
- How testing is done?
- What is its potential to find anything useful?
- Likely costs for tests/subsequent treatment if offered?
- Likelihood of finding incidental findings?
- Had you heard of whole genome sequencing before the study? Genetic testing?

3. **Now that you have been in the study for a while, are you still happy you joined the study?**

Probe:
- Why did you join the study?
- What has changed?
- Expectations versus reality
- How do you feel about waiting for your results? Potentially not getting results?

4. **What do you see as the benefits (if any) of whole genome sequencing?**

Probe:
- What do you hope to gain from this test (if anything)?
- What do you think the test will tell you?
- What types of information do you expect to learn (was there a family history)?

5. **Is there anything that worries you about it?**

Note for interviewer: Looking for examples of financial, emotional and practical drawbacks, like third party access to information, impact on insurance.

6. **Why did you decide to participate?**

Note for interviewer: Interested in rationale for participating, particularly in relation to any drawbacks the participant listed in Question 6.
Probe:
- To help medical research; learn about your genetics; help medical care; because I have been in another similar study

7. **In this study, you can choose to have all genetic information with medical relevance, or just cancer actionable information returned. Can you tell me why you chose {consent status}?**

Probe:
- Why; why not? (rationale for cancer results and nothing else)

8. **For the next few questions, I just want to shift focus a bit and ask you about conversations you have had about your decision to participate in this study. Who have you spoken to about this study?**

Probe for people they have spoken to:

- Why did you tell this person?
- How did you decide who to tell? (what factors were important in making that decision?)
- What did you say? What were the conversations like? How did the subject come up? How did you explain it? Were there differences in how you discussed your participation between immediate and distant relatives
- What were his/her/ their reactions?
- Were there particular aspects of the study your family/friend/HCP wanted to know about? Or things they did not want to know about?
- Did they decide to do anything differently after they heard about you having WGS? (e.g. seeking screening, testing etc)
- When did you talk to your relatives? (while making the decision or afterwards)
- Was it hard to talk about the study? Why or why not?
- How does your family usually talk about difficult things? Did you find it different, talking about WGS with them? Why or why not?
- What are some of your family dynamics or characteristics that influenced who you talked to about having WGS?
- What support would help you in having these conversations? What would make this conversation easier (online/written resources, a HCP talking to them/sending a letter)
- Did you tell anyone else about WGS? (e.g. friends, HCP, employer, in-laws)
    - If so, who?

Probe for people they are planning to speak to:

- Are you planning on sharing your decision to have WGS with your immediate/distant relatives?
    - Why/why not?
- Are there particular people in your family that you would struggle to talk to about having WGS? Why do you think it is difficult to talk to these people? (own and relatives' barriers)
- Are you planning on sharing your decision to have WGS with your healthcare professionals?

9. **Have you thought about who you might talk to about your results from the study? Why them?**

Probe:

- Are you planning on sharing your WGS results with your immediate/distant relatives? Which ones? (explore differences between immediate and distant relatives)
- Why/why not?
- Are there particular people in your family that you would struggle/find it hard to talk to about the results? Why do you think it will be difficult? (own and relatives' barriers)
- How will you decide who to tell? (what factors are important in making that decision?)
- How do you think that conversation will go? (explore differences between immediate and distant relatives) Are there any tools/support/resources you can think of that might make this discussion easier?
- What are some of your family dynamics or characteristics that would influence that conversation?
- Are you planning on sharing your results with your health care professionals? Which ones? Why/why not?
- Is there anyone else you are planning to tell? Who? (eg friends, employer, in-laws)

10. **Can you tell me what if feels like for you when you are experiencing uncertainty? How do you cope with uncertainty in your life? What sort of uncertainty are you experiencing about whole genome sequencing?**

Probe:

- Lots of people who undergo whole genome sequencing are uncertain about how their results may affect their insurance, are you unsure about this? How are you coping with that uncertainty?
- Lots of people who undergo whole genome sequencing are uncertain about how their results may affect their relatives, are you experiencing this uncertainty? How are you coping with that uncertainty?
- What is the hardest part to cope with?
- What would help you manage your uncertainty?

11. **In this study, you will only get results if the researchers find something important about your health? How do you feel about not knowing whether you will get test results? How are you coping with not being sure about whether you will get a result?**

Probe:

- How certain are you that the researchers will find something in the sequencing? How are you coping with that?
- With whole genome sequencing, if something is found, researchers won't be able to tell you if you will or will not get cancer in the future, they will only be able to give you an estimate of your risk. How will you cope with that indefinite result?
- How confident are you that the researchers understand the science enough to interpret what they find? How are you coping with that?
- How do you cope with the uncertainty around not knowing how and what they are doing, and when you will receive results? Are you expecting to hear something from the research team? (were they aware that no results may come)

12. **In this study, you will only be told about changes in genes that are important to your health, so this scenario will not happen to you in this study. But what do you think your reaction would be if you were told that changes in your genes had been found, but no-one knew what they meant? Do you think people should be told these sorts of results?**

13. **Do you think whole genome sequencing should be offered to all people with a family history of cancer?**

Probe:

- Why; why not?
- Is there anyone else that should be offered the test?

14. **Is there anything else you would like to say about whole genome sequencing? Do you have any advice about how this topic should be discussed with other people?**
