# Supplementary material for: Cancer Patient Experience of Uncertainty While Waiting for Genome Sequencing Results
Source: Front Psychol. 2021 Apr 22;12:647502. doi: 10.3389/fpsyg.2021.647502 (PMC8100530; doi:10.3389/fpsyg.2021.647502)
Supplement: Supplementary file 5 [file Data_Sheet_5.PDF]

RisC Study: 12 month follow up (T2) interview guide

Introduction:

Introduce self – working on the RisC study

Are you still ok to do this interview now?

Purpose - to discuss your experience of the study - no right or wrong ways of experiencing this, we are interested in your perspective

Will take no longer than 30 minutes - can stop the interview anytime you like. If you do not understand any of the questions, I will try my best to explain it.

Recording the interviews to help us remember and analyse everyone's responses; your recording will be anonymised and your identity will not be reported. Are you happy for me to record this interview?

Do you have any questions before we begin?

Questions:

1. **Tell me a bit about how the last 12 months have been going for you. Has anything about your participation in the RisC study changed since we last spoke?**

2. **Can you tell me what you remember about the study?**

3. **How are you feeling now about having whole genome sequencing done?**

Probe understanding of:
- How certain are you that the researchers will find something with the testing?
- How confident are you that the researchers will be able to interpret what they find?
- How are you coping with the uncertainty about not knowing what the researchers are doing and if and when you will receive your results?

4. **Now that you have been in the study for 12 months, what do you see as the benefits (if any) of whole genome sequencing? Have these changed for you over the 12 months?**

Probe:
- What do you hope to gain from this test (if anything)?
- What do you think the test will tell you?
- What types of information do you expect to learn (was there a family history)?
- Is there anything reassuring about whole genome sequencing for you?

5. **On reflection, is there anything that worries you about it?**

Note for interviewer: Looking for examples of financial, emotional and practical drawbacks, like third party access to information, impact on insurance.

6. **Are you still happy you joined the study?**

Probe:
- What has changed?
- Has the study met your expectations? (expectations versus reality)

7. **In this study, you will only be contacted if the testing shows important information about you. How do you feel about not knowing whether you will get test results? How are you coping?**

Probe:
- Are you expecting to hear something from the research team? (were they aware that no results may come)
- How do you feel about waiting for your results? Potentially not getting results?
- Tolerance of uncertainty - What could be done to mitigate anxiety? – information seeking, etc

8. **Over the past 12 months, have you made any cancer risk reduction changes to your lifestyle?**

Note for interviewer: Interested in behavior changes based on just family history versus WGS. Looking for understanding of what is modifiable and the likelihood of making changes.
Probe:
- For their cancer (family history), what lifestyle risk reduction options are there?
- What sort of changes have they made (if any)? – diet; exercise; alcohol, stress management; reproduction; surgery; increased screening

9. **Whole genome sequencing is looking to see if you are at increased risk of any cancers. Do you think you would change your lifestyle if you found you had an increased risk for cancer?**

   If yes, what?

   With whole genome sequencing, if the researchers find a result, they won't be able to tell you that you will or won't get another cancer or other illness in the future, they will only be able to give you an estimate of your risk. How do you think you will cope with that indefinite result?

10. **If you get a result, do you intend to share your result with your health professionals?**

Probe:
- Who? – GP; Oncologist; geneticist
- Why/why not?

11. **Do you plan to talk to your relatives about your test and the results?**

Probe:
- Why/why not?
- Which relatives?
- When? How?
- What would you say?
- How are you feeling about that conversation?
- Anticipated versus actual response?

12. Lots of people experience uncertainty when they are having genome sequencing. For example, uncertainty about how results will affect their insurance or how their results could impact their family members. What sort of uncertainty are you experiencing about whole genome sequencing?

13. What are your expecations of the study in the future?

    Note for interviewer: Interested in whether the participant sees this as clinical or research, and an understanding of there being no end-point to the study.

14. Is there anything else you would like to say about your participation in the study or whole genome sequencing?
