# Supplementary material for: Cancer Patient Experience of Uncertainty While Waiting for Genome Sequencing Results
Source: Front Psychol. 2021 Apr 22;12:647502. doi: 10.3389/fpsyg.2021.647502 (PMC8100530; doi:10.3389/fpsyg.2021.647502)
Supplement: Supplementary file 6 [file Data_Sheet_6.PDF]

Genetic Cancer Risk in the Young Study

Short title: Cancer Risk Study

Participant Questionnaire 3

As part of the Cancer Risk Study, we hope to better understand the impact of new genetic testing technologies on emotions and behaviour. To gain this understanding it is important for us to ask you about your experience at different times throughout your involvement in the Cancer Risk Study. The Cancer Risk Study involves a total of three questionnaires.

Thank you for completing Questionnaires 1 and 2. We are now inviting you to complete the third and final questionnaire for the Cancer Risk Study. In this questionnaire, we are asking about all possible responses to whole genome sequencing. Many of these may not be relevant to you, but it is important for us to get a clear picture of everyone's experiences.

We would greatly appreciate if you completed this questionnaire within the next 1-2 weeks. It will take about 20 minutes. Once completed the questionnaire can be submitted online or returned using the reply paid envelope provided.

Your responses will be kept confidential and your identity will not be revealed in any reports or presentations. Information you provide will not be shared with any health professionals involved in your care.

Participation in this study is voluntary and you can withdraw from the study at any time. If you would prefer not to participate in the study, please let us know by contacting the study coordinator (Mandy Ballinger, 02 9355 5806). Your participation (or non-participation) will not affect your relationship with any treating doctors, other health professionals or researchers involved with the Cancer Risk Study.

If you have any questions about filling in the questionnaire, or the study in general, please call the project officer Christine Napier on (02 9355 5839).

Participant number:

			--				

Date issued:

Date completed:

1. **People who are at high risk of cancer often worry about the possibility of developing cancer.**

   Most people who have had cancer worry about the possibility of a recurrence of the cancer. By recurrence we mean the possibility that the cancer will return or progress in the same place or in another part of your body.

   For each question please tick the box for the answer that best reflects how you felt in THE PAST MONTH.

a. **How often have you worried about the possibility of developing cancer / having a recurrence of cancer?**

0	1	2	3	4	5	6	7	8	9	10

None of the time — All of the time

b. **To what extent does worry about developing cancer / having a recurrence of cancer spill over or intrude on your thoughts and activities?**

0	1	2	3	4	5	6	7	8	9	10

Not at all — A great deal

c. **How emotionally upset or distressed have you been about the possibility of developing cancer / having a recurrence of cancer?**

0	1	2	3	4	5	6	7	8	9	10

Not at all — A great deal

For the next questions, compare yourself with someone who has the same risk of cancer, or the same cancer as you.

d. **Compared to an average person of the same age and gender as you, what do you think are your chances of developing cancer or having a recurrence of cancer?**

Much lower	Lower	Same	Higher	Much higher
❑	❑	❑	❑	❑

e. **If you were to express this as a number, or percentage, what would you say? Please place a vertical mark on the line below, where 0% = I am certain that I will not develop / have a recurrence of cancer; and 100% = I am certain that I will develop / have a recurrence of cancer.**

**0%**	**50%**	**100%**
**No chance of cancer progression or developing cancer**	**50-50 chance of progression or developing cancer**	**Will definitely progress or I will develop another cancer**

f. Compared to an average person of the same age and gender as you, what do you think are your chances of having a gene variant that puts you at increased cancer risk?

Much lower	Lower	Same	Higher	Much higher
❑	❑	❑	❑	❑

The next set of questions asks what you *think about* whole genome sequencing. Please tick one of the boxes to show how much you agree or disagree with the statement.

2. If I have whole genome sequencing:

	Strongly disagree	Disagree	Neither agree or disagree	Agree	Strongly agree
a. It is likely to provide information that would clarify how I could reduce my risk of (another) cancer in the future	❑	❑	❑	❑	❑
b. I may experience fear and distress when I find out the results	❑	❑	❑	❑	❑
c. It would make me feel like I had done everything I could to reduce my risk of a future cancer	❑	❑	❑	❑	❑
d. I may find it difficult to tell my family about the results	❑	❑	❑	❑	❑
e. I may find out about other gene variants that mean I and my family can plan and prepare for future risks of certain diseases	❑	❑	❑	❑	❑
f. If a gene variant is found, it would cost me a lot of money to get help to reduce my risk	❑	❑	❑	❑	❑
g. It would give me hope	❑	❑	❑	❑	❑
h. I may find out I have gene variants that no-one knows anything about	❑	❑	❑	❑	❑
i. It is unlikely anything would be found that would help me	❑	❑	❑	❑	❑

Please list any other *benefits* you think whole genome sequencing has:

____

____

Please list any other *drawbacks* you think whole genome sequencing has:

____

____

The questions below are about some specific responses you may have had while you wait for the results of the blood test (whole genome sequencing) that you had for this study.

3. Please indicate whether you have experienced each statement *never, rarely, sometimes,* or *often* IN THE PAST WEEK, by ticking the corresponding box.

	**Never**	**Rarely**	**Sometimes**	**Often**
a. Feeling upset about not receiving my whole genome sequencing test result	❑	❑	❑	❑
b. Feeling sad about not receiving my test result	❑	❑	❑	❑
c. Feeling anxious or nervous about not receiving my test result	❑	❑	❑	❑
d. Feeling relieved about not receiving my test result	❑	❑	❑	❑
e. Feeling happy about not receiving my test result	❑	❑	❑	❑
f. Feeling a loss of control	❑	❑	❑	❑
g. Having problems enjoying my life while waiting for my test result	❑	❑	❑	❑
h. Worrying about my risk of cancer developing	❑	❑	❑	❑
i. Being uncertain about what my test result will mean about my cancer risk	❑	❑	❑	❑
j. Being uncertain about what my test result will mean for my child(ren) and / or family's cancer risk	❑	❑	❑	❑
k. Having difficulty making decisions about my cancer risk	❑	❑	❑	❑
l. Understanding clearly my choices for cancer risk reduction	❑	❑	❑	❑
m. Feeling frustrated that there are no definite cancer risk reduction options for me yet	❑	❑	❑	❑
n. Thinking about not receiving my test result has affected my work or family life	❑	❑	❑	❑
o. Feeling concerned about how my test result will affect my insurance status	❑	❑	❑	❑
p. Having difficulty talking about my cancer risk with family members	❑	❑	❑	❑
q. Feeling that my family has been supportive during the whole genome sequencing process	❑	❑	❑	❑
r. Feeling satisfied with family communication while waiting for my test result	❑	❑	❑	❑
s. Worrying that the whole genome sequencing process has brought about conflict within my family	❑	❑	❑	❑

If you have children, please answer the next question. Otherwise, please go to question 5.

4. **Please indicate whether you have experienced each statement *never, rarely, sometimes,* or *often* IN THE PAST WEEK, by ticking the corresponding box.**

	Never	Rarely	Sometimes	Often
a. Worrying about the possibility of my children getting cancer	❑	❑	❑	❑
b. Feeling guilty about possibly passing on the disease risk to my child(ren)	❑	❑	❑	❑

5. **Please indicate whether you have experienced each statement *never, rarely, sometimes,* or *often* IN THE PAST WEEK, by ticking the corresponding box.**

	Never	Rarely	Sometimes	Often
a. Feeling that not receiving the whole genome sequencing test result has made it harder to cope with my cancer risk	❑	❑	❑	❑
b. Feeling that not receiving the whole genome sequencing test result has made it easier to cope with my cancer risk	❑	❑	❑	❑

6. **The next question asks about whether you worry about the results of your *whole genome sequencing and what they will mean for you*.**

   Mark a response for each item, indicating how frequently these comments were true for you DURING THE PAST SEVEN DAYS. If they did not occur during that time, please mark the "not at all" column.

	Not at all	Rarely	Sometimes	Often
a. I thought about it (my whole genome sequencing results) when I didn't mean to	❑	❑	❑	❑
b. I avoided letting myself get upset when I thought about it or was reminded of it	❑	❑	❑	❑
c. I tried to remove it from memory	❑	❑	❑	❑
d. I had trouble falling asleep or staying asleep, because of pictures or thoughts about it that came into my mind	❑	❑	❑	❑
e. I had waves of strong feelings about it	❑	❑	❑	❑
f. I had dreams about it	❑	❑	❑	❑
g. I stayed away from reminders of it	❑	❑	❑	❑
h. I felt as if it hadn't happened or it wasn't real	❑	❑	❑	❑
i. I tried not to talk about it	❑	❑	❑	❑
j. Pictures about it popped into my mind	❑	❑	❑	❑
k. Other things kept making me think about it	❑	❑	❑	❑

	Not at all	Rarely	Sometimes	Often
l. I was aware that I still had a lot of feelings about it, but I didn't deal with them	❑	❑	❑	❑
m. I tried not to think about it	❑	❑	❑	❑
n. Any reminder brought back feelings about it	❑	❑	❑	❑
o. My feelings about it were kind of numb	❑	❑	❑	❑

The next questions ask you about your emotions.

7. Choose the best response to describe how you have been feeling OVER THE LAST WEEK. Don't take too long over your replies; your immediate reaction will probably be more accurate than a long thought-out response.

a. I feel tense or 'wound up':

❑ Most of the time
❑ A lot of the time
❑ From time to time, occasionally
❑ Not at all

b. I still enjoy the things I used to enjoy:

❑ Definitely as much
❑ Not quite so much
❑ Only a little
❑ Hardly at all

c. I get a sort of frightened feeling as if something awful is about to happen:

❑ Very definitely and quite badly
❑ Yes, but not too badly
❑ A little, but it doesn't worry me
❑ Not at all

d. I can laugh and see the funny side of things:

❑ As much as I always could
❑ Not quite so much now
❑ Definitely not so much now
❑ Not at all

e. Worrying thoughts go through my mind:

❑ A great deal of the time
❑ A lot of the time
❑ From time to time, but not too often
❑ Only occasionally

f. I feel cheerful:

❑ Not at all
❑ Not often
❑ Sometimes
❑ Most of the time

g. I can sit at ease and feel relaxed:

❑ Definitely
❑ Usually
❑ Not often
❑ Not at all

h. I feel as if I am slowed down:

❑ Nearly all the time
❑ Very often
❑ Sometimes
❑ Not at all

i. I get a sort of frightened feeling like 'butterflies' in the stomach:

❑	Not at all
❑	Occasionally
❑	Quite often
❑	Very often

j. I have lost interest in my appearance:

❑	Definitely
❑	I don't take as much care as I should
❑	I may not take quite as much care
❑	I take just as much care as ever

k. I feel restless as if I have to be on the move:

❑	Very much indeed
❑	Quite a lot
❑	Not very much
❑	Not at all

l. I look forward with enjoyment to things:

❑	As much as ever I did
❑	Rather less than I used to
❑	Definitely less than I used to
❑	Hardly at all

m. I get sudden feelings of panic:

❑	Very often indeed
❑	Quite often
❑	Not very often
❑	Not at all

n. I can enjoy a good book or radio or TV programme:

❑	Often
❑	Sometimes
❑	Not often
❑	Very seldom

8. Answer this question according to how much you agree or disagree with each statement RIGHT NOW.

	**Strongly disagree**	**Disagree**	**Agree**	**Strongly agree**
a. I have a positive outlook toward life	❑	❑	❑	❑
b. I have short and / or long range goals	❑	❑	❑	❑
c. I feel all alone	❑	❑	❑	❑
d. I can see possibilities in the midst of difficulties	❑	❑	❑	❑
e. I have a faith that gives me comfort	❑	❑	❑	❑
f. I feel scared about my future	❑	❑	❑	❑
g. I can recall happy / joyful times	❑	❑	❑	❑
h. I have deep inner strength	❑	❑	❑	❑
i. I am able to give and receive caring / love	❑	❑	❑	❑
j. I have a sense of direction	❑	❑	❑	❑
k. I believe that each day has potential	❑	❑	❑	❑
l. I feel my life has value and worth	❑	❑	❑	❑

The next two questions ask you about how happy you are with the decision you made to have whole genome sequencing.

9. Please show how strongly you agree or disagree with these statements by ticking the box that best fits your view about your decision to have whole genomic sequencing.

	**Strongly disagree**	**Disagree**	**Neither agree or disagree**	**Agree**	**Strongly agree**
a. It was the right decision	❑	❑	❑	❑	❑
b. I regret the choice that was made	❑	❑	❑	❑	❑
c. I would go for the same choice if I had to do it over again	❑	❑	❑	❑	❑
d. The choice did me a lot of harm	❑	❑	❑	❑	❑
e. The decision was a wise one	❑	❑	❑	❑	❑

10. Considering your decision to <u>have whole genome sequencing</u>, please indicate to what extent each statement is true for you AT THIS TIME.

	**Strongly disagree**	**Disagree**	**Neither agree or disagree**	**Agree**	**Strongly agree**
a. I am satisfied that I was adequately informed about the issues important to my decision	❑	❑	❑	❑	❑
b. The decision I made was the best decision possible for me personally	❑	❑	❑	❑	❑
c. I am satisfied that my decision was consistent with my personal values	❑	❑	❑	❑	❑
d. I expect to successfully carry out the decision I made	❑	❑	❑	❑	❑
e. I am satisfied that this was my decision to make	❑	❑	❑	❑	❑
f. I am satisfied with my decision	❑	❑	❑	❑	❑

11. Under each heading, please tick the ONE box that best describes your health TODAY.

a. MOBILITY

❑	I have no problems with walking around
❑	I have slight problems with walking around
❑	I have moderate problems with walking around
❑	I have severe problems with walking around
❑	I am unable to walk around

b. PERSONAL CARE

❑	I have no problems with washing or dressing myself
❑	I have slight problems with washing or dressing myself
❑	I have moderate problems with washing or dressing myself
❑	I have severe problems with washing or dressing myself
❑	I am unable to wash or dress myself

c. USUAL ACTIVITIES (e.g. work, study, housework, family or leisure activities)

❑	I have no problems doing my usual activities
❑	I have slight problems doing my usual activities
❑	I have moderate problems doing my usual activities
❑	I have severe problems doing my usual activities
❑	I am unable to do my usual activities

d. PAIN / DISCOMFORT

❑	I have no pain or discomfort
❑	I have slight pain or discomfort
❑	I have moderate pain or discomfort
❑	I have severe pain or discomfort
❑	I have extreme pain or discomfort

e. ANXIETY / DEPRESSION

❑	I am not anxious or depressed
❑	I am slightly anxious or depressed
❑	I am moderately anxious or depressed
❑	I am severely anxious or depressed
❑	I am extremely anxious or depressed

12. We would like to know how good or bad your health is TODAY.

- This scale is numbered from 0 to 100
- 100 means the <u>best</u> health you can imagine,
  0 means the <u>worst</u> health you can imagine
- Mark an X on the scale to indicate how your health is **TODAY**
- Now, please write the number you marked on the scale in the box below

YOUR HEALTH TODAY = ☐

The best health
you can imagine

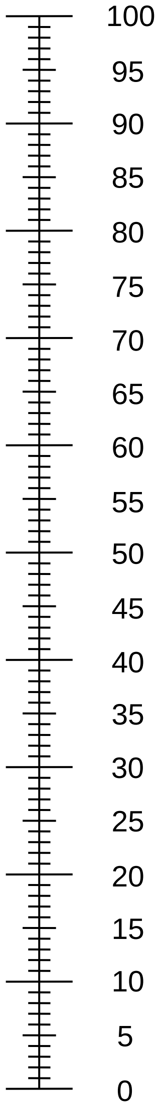

The worst health
you can imagine

13. How much energy do you have to do the things you want to do? I am

- ❑ Always full of energy
- ❑ Usually full of energy
- ❑ Occasionally energetic
- ❑ Usually tired and lacking energy
- ❑ Always tired and lacking energy

14. How often do you feel socially excluded or left out?

- ❑ Never
- ❑ Rarely
- ❑ Sometimes
- ❑ Often
- ❑ Always

15. How easy or difficult is it for you to get around by yourself outside your place of residence (e.g. to go shopping, visiting)?

- ❑ Getting around is enjoyable and easy
- ❑ I have no difficulty getting around outside my place of residence
- ❑ A little difficulty
- ❑ Moderate difficulty
- ❑ A lot of difficulty
- ❑ I cannot get around unless somebody is there to help me

16. Does your health affect your role in your community (e.g. residential, sporting, church or cultural activities)?

- ❑ My role in the community is unaffected by my health
- ❑ There are some parts of my community role I cannot carry out
- ❑ There are many parts of my community role I cannot carry out
- ❑ I cannot carry out any part of my community role

17. How often do you feel sad?

- ❑ Never
- ❑ Rarely
- ❑ Some of the time
- ❑ Usually
- ❑ Nearly all the time

18. How often do you experience serious pain? I experience it

- ❑ Very rarely
- ❑ Less than once a week
- ❑ Three to four times a week
- ❑ Most of the time

19. How much confidence do you have in yourself?

- ❑ Complete confidence
- ❑ A lot
- ❑ A moderate amount
- ❑ A little
- ❑ None at all

20. Do you normally feel calm and tranquil or agitated? I am

- ❑ Always calm and tranquil
- ❑ Usually calm and tranquil
- ❑ Sometimes calm and tranquil, sometimes agitated
- ❑ Usually agitated
- ❑ Always agitated

21. Does your health affect your relationship with your family?

- ❑ My role in the family is unaffected by my health
- ❑ There are some parts of my family role I cannot carry out
- ❑ There are many parts of my family role I cannot carry out
- ❑ I cannot carry out any part of my family role

22. How satisfying are your close relationships (family and friends)?

❑	Very satisfying
❑	Satisfying
❑	Neither satisfying nor dissatisfying
❑	Dissatisfying
❑	Unpleasant
❑	Very unpleasant

23. How well do you communicate with others (talking, signing, texting, being understood by others and understanding them)?

❑	I have no trouble being understood
❑	I have some difficulty being understood by people who do not know me
❑	I am understood only by people who know me
❑	I cannot adequately communicate with others

24. How often do you have trouble sleeping?

❑	Never
❑	Almost never
❑	Sometimes
❑	Often
❑	All the time

25. How often do you feel worthless?

❑	Never
❑	Almost never
❑	Sometimes
❑	Usually
❑	Always

26. How often do you feel angry?

❑	Never
❑	Almost never
❑	Sometimes
❑	Often
❑	All the time

27. How easy or difficult is it for you to move around (using any aids or equipment you need e.g. a wheelchair, frame or stick)?

❑	I am very mobile
❑	I have no difficulty with mobility
❑	I have some difficulty with mobility (for example, going uphill)
❑	I have difficulty with mobility. I can go short distances only.
❑	I have a lot of difficulty with mobility. I need someone to help me.
❑	I am bedridden

28. Do you ever feel like hurting yourself?

❑	Never
❑	Rarely
❑	Sometimes
❑	Often
❑	All the time

29. How enthusiastic do you feel?

❑	Extremely
❑	Very
❑	Somewhat
❑	Not much
❑	Not at all

30. How often did you feel worried in the last seven days?

❑	Never
❑	Occasionally
❑	Sometimes
❑	Often
❑	All the time

31. How difficult is it for you to wash, toilet, dress yourself, eat or care for your appearance?

❑	These things are very easy for me to do
❑	I have no real difficulty in doing these things
❑	I find some of these things difficult, but I manage to do them on my own
❑	Many of these things are difficult, and I need help to do them
❑	I cannot do these things by myself at all

32. How often do you feel happy?

❑	All the time
❑	Mostly
❑	Sometimes
❑	Almost never
❑	Never

33. How much do you feel you can cope with life's problems?

❑	Completely
❑	Mostly
❑	Partly
❑	Very little
❑	Not at all

34. How much pain or discomfort do you experience:

❑	None at all
❑	I have moderate pain
❑	I suffer from severe pain
❑	I suffer unbearable pain

35. How much do you enjoy your close relationships (family and friends)?

❑	Immensely
❑	A lot
❑	A little
❑	Not much
❑	I hate it

36. How often does pain interfere with your usual activities?

❑	Never
❑	Rarely
❑	Sometimes
❑	Often
❑	Always

37. How often do you feel pleasure?

❑	Always
❑	Usually
❑	Sometimes
❑	Almost never
❑	Never

38. How much of a burden do you feel you are to other people?

❑	Not at all
❑	A little
❑	A moderate amount
❑	A lot
❑	Totally

39. How content are you with your life?

❑	Extremely
❑	Mainly
❑	Moderately
❑	Slightly
❑	Not at all

40. How well can you see (using your glasses or contact lenses if they are needed)?

❑	I have excellent sight
❑	I see normally
❑	I have some difficulty seeing things sharply (e.g. small print, objects in the distance, or watching television)
❑	I have a lot of difficulty seeing sharply
❑	I only see general shapes
❑	I am completely blind

41. How often do you feel in control of your life?

❑	Always
❑	Mostly
❑	Sometimes
❑	Only occasionally
❑	Never

42. How much help do you need with jobs around your place of residence (e.g. preparing food, cleaning, gardening)?

❑	I can do all these tasks very easily without any help
❑	I can do these tasks relatively easily without help
❑	I can do these tasks only very slowly without help
❑	I cannot do most of these tasks unless I have help
❑	I can do none of these tasks by myself

43. How often do you feel socially isolated?

❑	Never
❑	Rarely
❑	Sometimes
❑	Often
❑	Always

44. How well can you hear (using your hearing aid if needed)?

❑	I have excellent hearing
❑	I hear normally
❑	I have some difficulty hearing or I do not hear clearly (e.g. when there is background noise)
❑	I have difficulty hearing things clearly. Often I do not understand what is said. I usually do not take part in conversations because I cannot hear what is said.
❑	I hear very little
❑	I am completely deaf

45. How often do you feel depressed?

❑	Never
❑	Almost never
❑	Sometimes
❑	Often
❑	Very often
❑	All the time

46. How happy are you with your close and intimate relationships?

❑	Very happy
❑	Generally happy
❑	Neither happy nor unhappy
❑	Generally unhappy
❑	Very unhappy

47. How often did you feel in despair in the last seven days?

❑	Never
❑	Occasionally
❑	Sometimes
❑	Often
❑	All the time

THANK YOU FOR COMPLETING THIS QUESTIONNAIRE
