# Supplementary material for: Cancer Patient Experience of Uncertainty While Waiting for Genome Sequencing Results
Source: Front Psychol. 2021 Apr 22;12:647502. doi: 10.3389/fpsyg.2021.647502 (PMC8100530; doi:10.3389/fpsyg.2021.647502)
Supplement: Supplementary file 7 [file Data_Sheet_7.PDF]

Genetic Cancer Risk in the Young Study

Short title: Cancer Risk Study

Participant Questionnaire 2

As part of the Cancer Risk Study, we hope to better understand the impact of new genetic testing technologies on emotions and behaviour. To gain this understanding it is important for us to ask you about your experience at different times throughout your involvement in the Cancer Risk Study. The Cancer Risk Study involves a total of three questionnaires.

Thank you for completing Questionnaire 1. We are now inviting you to complete this questionnaire (Questionnaire 2). In this questionnaire, we are asking about all possible responses to whole genome sequencing. Many of these may not be relevant to you, but it is important for us to get a clear picture of everyone's experiences.

We would greatly appreciate if you completed this questionnaire within the next 1-2 weeks. It will take about 20 minutes. Once completed the questionnaire can be submitted online or returned using the reply paid envelope provided.

Your responses will be kept confidential and your identity will not be revealed in any reports or presentations. Information you provide will not be shared with any health professionals involved in your care.

Participation in this study is voluntary and you can withdraw from the study at any time. If you would prefer not to participate in the study, please let us know by contacting the study coordinator (Mandy Ballinger, 02 9355 5806). Your participation (or non-participation) will not affect your relationship with any treating doctors, other health professionals or researchers involved with the Cancer Risk Study.

If you have any questions about filling in the questionnaire, or the study in general, please call the project officer Christine Napier on (02 9355 5839).

Participant number:

			--				

Date issued:

Date completed:

1. **People who are at high risk of cancer often worry about the possibility of developing cancer.**

   Most people who have had cancer worry about the possibility of a recurrence of the cancer. By recurrence we mean the possibility that the cancer will return or progress in the same place or in another part of your body.

   For each question please tick the box for the answer that best reflects how you felt in THE PAST MONTH.

a. How often have you worried about the possibility of developing cancer / having a recurrence of cancer?

0	1	2	3	4	5	6	7	8	9	10

None of the time … All of the time

b. To what extent does worry about developing cancer / having a recurrence of cancer spill over or intrude on your thoughts and activities?

0	1	2	3	4	5	6	7	8	9	10

Not at all … A great deal

c. How emotionally upset or distressed have you been about the possibility of developing cancer / having a recurrence of cancer?

0	1	2	3	4	5	6	7	8	9	10

Not at all … A great deal

For the next questions, compare yourself with someone who has the same risk of cancer, or the same cancer as you.

d. Compared to an average person of the same age and gender as you, what do you think are your chances of developing cancer or having a recurrence of cancer?

Much lower	Lower	Same	Higher	Much higher
❑	❑	❑	❑	❑

e. If you were to express this as a number, or percentage, what would you say? Please place a vertical mark on the line below, where 0% = I am certain that I will not develop / have a recurrence of cancer; and 100% = I am certain that I will develop / have a recurrence of cancer.

**0%**	**50%**	**100%**
**No chance of cancer progression or developing cancer**	**50-50 chance of progression or developing cancer**	**Will definitely progress or I will develop another cancer**

f. Compared to an average person of the same age and gender as you, what do you think are your chances of having a gene variant that puts you at increased cancer risk?

Much lower	Lower	Same	Higher	Much higher
❑	❑	❑	❑	❑

The next set of questions asks what you *think about* whole genome sequencing. Please tick one of the boxes to show how much you agree or disagree with the statement.

2. If I have whole genome sequencing:

	Strongly disagree	Disagree	Neither agree or disagree	Agree	Strongly agree
a. It is likely to provide information that would clarify how I could reduce my risk of (another) cancer in the future	❑	❑	❑	❑	❑
b. I may experience fear and distress when I find out the results	❑	❑	❑	❑	❑
c. It would make me feel like I had done everything I could to reduce my risk of a future cancer	❑	❑	❑	❑	❑
d. I may find it difficult to tell my family about the results	❑	❑	❑	❑	❑
e. I may find out about other gene variants that mean I and my family can plan and prepare for future risks of certain diseases	❑	❑	❑	❑	❑
f. If a gene variant is found, it would cost me a lot of money to get help to reduce my risk	❑	❑	❑	❑	❑
g. It would give me hope	❑	❑	❑	❑	❑
h. I may find out I have gene variants that no-one knows anything about	❑	❑	❑	❑	❑
i. It is unlikely anything would be found that would help me	❑	❑	❑	❑	❑

Please list any other *benefits* you think whole genome sequencing has:

____

____

Please list any other *drawbacks* you think whole genome sequencing has:

____

____

The questions below are about some specific responses you may have had while you wait for the results of the blood test (whole genome sequencing) that you had for this study.

3. Please indicate whether you have experienced each statement *never, rarely, sometimes,* or *often* IN THE PAST WEEK, by ticking the corresponding box.

	**Never**	**Rarely**	**Sometimes**	**Often**
a. Feeling upset about not receiving my whole genome sequencing test result	❑	❑	❑	❑
b. Feeling sad about not receiving my test result	❑	❑	❑	❑
c. Feeling anxious or nervous about not receiving my test result	❑	❑	❑	❑
d. Feeling relieved about not receiving my test result	❑	❑	❑	❑
e. Feeling happy about not receiving my test result	❑	❑	❑	❑
f. Feeling a loss of control	❑	❑	❑	❑
g. Having problems enjoying my life while waiting for my test result	❑	❑	❑	❑
h. Worrying about my risk of cancer developing	❑	❑	❑	❑
i. Being uncertain about what my test result will mean about my cancer risk	❑	❑	❑	❑
j. Being uncertain about what my test result will mean for my child(ren) and / or family's cancer risk	❑	❑	❑	❑
k. Having difficulty making decisions about my cancer risk	❑	❑	❑	❑
l. Understanding clearly my choices for cancer risk reduction	❑	❑	❑	❑
m. Feeling frustrated that there are no definite cancer risk reduction options for me yet	❑	❑	❑	❑
n. Thinking about not receiving my test result has affected my work or family life	❑	❑	❑	❑
o. Feeling concerned about how my test result will affect my insurance status	❑	❑	❑	❑
p. Having difficulty talking about my cancer risk with family members	❑	❑	❑	❑
q. Feeling that my family has been supportive during the whole genome sequencing process	❑	❑	❑	❑
r. Feeling satisfied with family communication while waiting for my test result	❑	❑	❑	❑
s. Worrying that the whole genome sequencing process has brought about conflict within my family	❑	❑	❑	❑

If you have children, please answer the next question. Otherwise, please go to question 5.

4. **Please indicate whether you have experienced each statement *never, rarely, sometimes,* or *often* IN THE PAST WEEK, by ticking the corresponding box.**

	**Never**	**Rarely**	**Sometimes**	**Often**
a. Worrying about the possibility of my children getting cancer	❑	❑	❑	❑
b. Feeling guilty about possibly passing on the disease risk to my child(ren)	❑	❑	❑	❑

5. **Please indicate whether you have experienced each statement *never, rarely, sometimes,* or *often* IN THE PAST WEEK, by ticking the corresponding box.**

	**Never**	**Rarely**	**Sometimes**	**Often**
a. Feeling that not receiving the whole genome sequencing test result has made it harder to cope with my cancer risk	❑	❑	❑	❑
b. Feeling that not receiving the whole genome sequencing test result has made it easier to cope with my cancer risk	❑	❑	❑	❑

6. **The next question asks about whether you worry about the results of your *whole genome sequencing and what they will mean for you*.**

   Mark a response for each item, indicating how frequently these comments were true for you DURING THE PAST SEVEN DAYS. If they did not occur during that time, please mark the "not at all" column.

	**Not at all**	**Rarely**	**Sometimes**	**Often**
a. I thought about it (my whole genome sequencing results) when I didn't mean to	❑	❑	❑	❑
b. I avoided letting myself get upset when I thought about it or was reminded of it	❑	❑	❑	❑
c. I tried to remove it from memory	❑	❑	❑	❑
d. I had trouble falling asleep or staying asleep, because of pictures or thoughts about it that came into my mind	❑	❑	❑	❑
e. I had waves of strong feelings about it	❑	❑	❑	❑
f. I had dreams about it	❑	❑	❑	❑
g. I stayed away from reminders of it	❑	❑	❑	❑
h. I felt as if it hadn't happened or it wasn't real	❑	❑	❑	❑
i. I tried not to talk about it	❑	❑	❑	❑
j. Pictures about it popped into my mind	❑	❑	❑	❑
k. Other things kept making me think about it	❑	❑	❑	❑

	Not at all	Rarely	Sometimes	Often
l. I was aware that I still had a lot of feelings about it, but I didn't deal with them	❑	❑	❑	❑
m. I tried not to think about it	❑	❑	❑	❑
n. Any reminder brought back feelings about it	❑	❑	❑	❑
o. My feelings about it were kind of numb	❑	❑	❑	❑

The next questions ask you about your emotions.

7. Choose the best response to describe how you have been feeling OVER THE LAST WEEK. Don't take too long over your replies; your immediate reaction will probably be more accurate than a long thought-out response.

a. I feel tense or 'wound up':

❑	Most of the time
❑	A lot of the time
❑	From time to time, occasionally
❑	Not at all

b. I still enjoy the things I used to enjoy:

❑	Definitely as much
❑	Not quite so much
❑	Only a little
❑	Hardly at all

c. I get a sort of frightened feeling as if something awful is about to happen:

❑	Very definitely and quite badly
❑	Yes, but not too badly
❑	A little, but it doesn't worry me
❑	Not at all

d. I can laugh and see the funny side of things:

❑	As much as I always could
❑	Not quite so much now
❑	Definitely not so much now
❑	Not at all

e. Worrying thoughts go through my mind:

❑	A great deal of the time
❑	A lot of the time
❑	From time to time, but not too often
❑	Only occasionally

f. I feel cheerful:

❑	Not at all
❑	Not often
❑	Sometimes
❑	Most of the time

g. I can sit at ease and feel relaxed:

❑	Definitely
❑	Usually
❑	Not often
❑	Not at all

h. I feel as if I am slowed down:

❑	Nearly all the time
❑	Very often
❑	Sometimes
❑	Not at all

i. I get a sort of frightened feeling like 'butterflies' in the stomach:

❑	Not at all
❑	Occasionally
❑	Quite often
❑	Very often

j. I have lost interest in my appearance:

❑	Definitely
❑	I don't take as much care as I should
❑	I may not take quite as much care
❑	I take just as much care as ever

k. I feel restless as if I have to be on the move:

❑	Very much indeed
❑	Quite a lot
❑	Not very much
❑	Not at all

l. I look forward with enjoyment to things:

❑	As much as ever I did
❑	Rather less than I used to
❑	Definitely less than I used to
❑	Hardly at all

m. I get sudden feelings of panic:

❑	Very often indeed
❑	Quite often
❑	Not very often
❑	Not at all

n. I can enjoy a good book or radio or TV programme:

❑	Often
❑	Sometimes
❑	Not often
❑	Very seldom

8. Answer this question according to how much you agree or disagree with each statement RIGHT NOW.

	Strongly disagree	Disagree	Agree	Strongly agree
a. I have a positive outlook toward life	❑	❑	❑	❑
b. I have short and / or long range goals	❑	❑	❑	❑
c. I feel all alone	❑	❑	❑	❑
d. I can see possibilities in the midst of difficulties	❑	❑	❑	❑
e. I have a faith that gives me comfort	❑	❑	❑	❑
f. I feel scared about my future	❑	❑	❑	❑
g. I can recall happy / joyful times	❑	❑	❑	❑
h. I have deep inner strength	❑	❑	❑	❑
i. I am able to give and receive caring / love	❑	❑	❑	❑
j. I have a sense of direction	❑	❑	❑	❑
k. I believe that each day has potential	❑	❑	❑	❑
l. I feel my life has value and worth	❑	❑	❑	❑

The last questions ask you about how happy you are with the decision you made to have whole genome sequencing as part of a research study.

9. Please show how strongly you agree or disagree with these statements by ticking the box which best fits your view about your decision to have whole genome sequencing.

	Strongly disagree	Disagree	Neither agree or disagree	Agree	Strongly agree
a. It was the right decision	❑	❑	❑	❑	❑
b. I regret the choice that was made	❑	❑	❑	❑	❑
c. I would go for the same choice if I had to do it over again	❑	❑	❑	❑	❑
d. The choice did me a lot of harm	❑	❑	❑	❑	❑
e. The decision was a wise one	❑	❑	❑	❑	❑

10. Considering your decision to have whole genome sequencing, please indicate to what extent each statement is true for you AT THIS TIME.

	Strongly disagree	Disagree	Neither agree or disagree	Agree	Strongly agree
a. I am satisfied that I was adequately informed about the issues important to my decision	❑	❑	❑	❑	❑
b. The decision I made was the best decision possible for me personally	❑	❑	❑	❑	❑
c. I am satisfied that my decision was consistent with my personal values	❑	❑	❑	❑	❑
d. I expect to successfully carry out the decision I made	❑	❑	❑	❑	❑
e. I am satisfied that this was my decision to make	❑	❑	❑	❑	❑
f. I am satisfied with my decision	❑	❑	❑	❑	❑

THANK YOU FOR COMPLETING THIS QUESTIONNAIRE
